# Arnicolide D induces endoplasmic reticulum stress-mediated oncosis via ATF4 and CHOP in hepatocellular carcinoma cells

**DOI:** 10.1038/s41420-024-01911-w

**Published:** 2024-03-12

**Authors:** Yu-Shan Lin, Zhiwei Sun, Li-Sha Shen, Rui-Hong Gong, Jia-Wen Chen, Yanfeng Xu, Haiyang Yu, Sibao Chen, Guo-Qing Chen

**Affiliations:** 1 State Key Laboratory of Chinese Medicine and Molecular Pharmacology (Incubation), The Hong Kong Polytechnic University Shenzhen Research Institute, Shenzhen, 518057 China; 2 Chongqing Academy of Chinese Materia Medica, Chongqing, 400065 China; 3 Department of Food Science and Nutrition, The Hong Kong Polytechnic University, Hung Hom, 999077 Hong Kong China; 4 Institute of Medicinal Plant Development, Chinese Academy of Medical Sciences and Peking Union Medical College, 100193 Beijing, China; 5 Shanghai Municipal Hospital of Traditional Chinese Medicine, Shanghai, 200071 China; 6 Key Laboratory of Pharmacology of Traditional Chinese Medical Formulae, Ministry of Education, and State Key Laboratory of Component-Based Chinese Medicine, Tianjin University of Traditional Chinese Medicine, Tianjin, 301617 China; 7 Research Centre for Chinese Medicine Innovation, The Hong Kong Polytechnic University, Hung Hom, 999077 Hong Kong China

**Keywords:** Natural products, Mechanism of action

## Abstract

Endoplasmic reticulum (ER) stress can trigger various cell death mechanisms beyond apoptosis, providing promise in cancer treatment. Oncosis, characterized by cellular swelling and increased membrane permeability, represents a non-apoptotic form of cell death. In our study, we discovered that Arnicolide D (AD), a natural sesquiterpene lactone compound, induces ER stress-mediated oncosis in hepatocellular carcinoma (HCC) cells, and this process is reactive oxygen species (ROS)-dependent. Furthermore, we identified the activation of the PERK-eIF2α-ATF4-CHOP pathway during ER stress as a pivotal factor in AD-induced oncosis. Notably, the protein synthesis inhibitor cycloheximide (CHX) was found to effectively reverse AD-induced oncosis, suggesting ATF4 and CHOP may hold crucial roles in the induction of oncosis by AD. These proteins play a vital part in promoting protein synthesis during ER stress, ultimately leading to cell death. Subsequent studies, in where we individually or simultaneously knocked down ATF4 and CHOP in HCC cells, provided further confirmation of their indispensable roles in AD-induced oncosis. Moreover, additional animal experiments not only substantiated AD’s ability to inhibit HCC tumor growth but also solidified the essential role of ER stress-mediated and ROS-dependent oncosis in AD’s therapeutic potential. In summary, our research findings strongly indicate that AD holds promise as a therapeutic agent for HCC by its ability to induce oncosis.

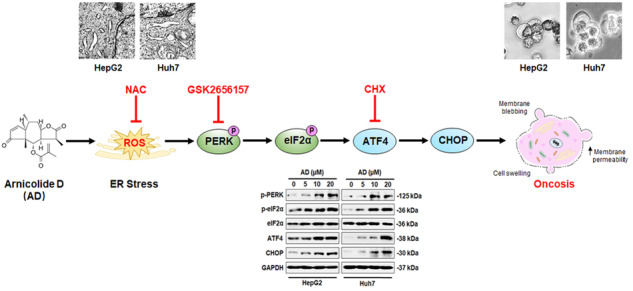

## Introduction

The endoplasmic reticulum (ER) is a significant cytoplasmic organelle in eukaryotic cells responsible for protein synthesis, folding, maturation, and translocation [1]. Disruptions to protein homeostasis, whether arising from environmental, physiological, or pathological factors, have the potential to induce ER stress [2]. Initially, ER stress serves as an adaptive cellular response, aiming to shield the cell from damage and dysfunction [3]. Notably, cancer cells heavily depend on an elevated ER stress response to manage misfolded proteins and sustain rapid growth [4]. This adaptive mechanism aids in restoring homeostasis in cancer cells, fostering an environment conducive to their survival and proliferation [5]. However, prolonged ER stress can trigger maladaptive responses, ultimately resulting in cell death [6]. Consequently, recognizing the dual role of ER stress in cancer development, there has been a shifting towards targeting and inducing persistent ER stress-mediated cell death as an innovative anticancer strategy [7].

It is widely acknowledged that cell death can manifest in various forms, with apoptosis emerging as one of the earliest and extensively studied types. Notably, ER stress has emerged as a key initiator of cell death, primarily through the induction of apoptosis. Several compounds have demonstrated their anticancer efficacy by inducing apoptosis through ER stress mechanisms [8, 9]. Intriguingly, the influence of ER stress extends beyond apoptosis, encompassing a range of non-apoptotic cell death pathways such as necroptosis [10], ferroptosis [11], and oncosis [12]. This revelation positions ER stress-mediated non-apoptotic cell death as an enticing strategy for interventions in anticancer approaches [13].

Oncosis, a non-apoptotic form of cell death, has garnered considerable attention. Coined from the Greek word for swelling, oncosis was first proposed in 1910 [14]. In contrast to apoptosis, which involves cell shrinkage, smooth-surfaced protuberances, and chromatin condensation, oncosis is characterized by cellular swelling and increased membrane permeability [15]. This distinction makes oncosis a promising avenue for anticancer research and therapy. Various compounds, including aspirin [16], fluopsin C [17], berberine [18], and artesunate [19], have been identified as oncosis inducers.

**Fig. 1:**
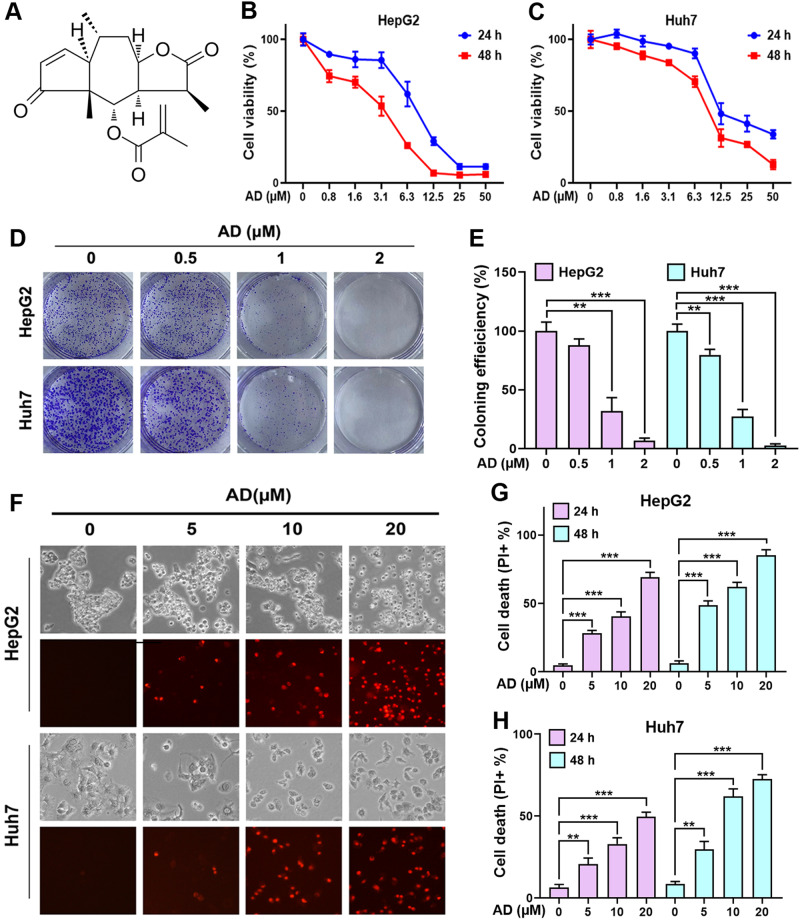
AD exhibited anticancer activity against HCC in vitro. **A** Chemical structure of AD. **B** HepG2 and **C** Huh7 cells were treated with the indicated concentration of AD, and their cell viability was assessed using MTT separately at 24 h and 48 h. **D** Colony formation of cells treated with the indicated concentration of AD, and **E** the impact on colony formation efficiency. **F** Induced cell death in cells treated with the indicated concentration of AD for 24 h. The upper panel displays phase-contrast images and the lower panel exhibits PI staining to identify dead cells. Scale bar = 100 μm. **G** HepG2 and **H** Huh7 cells were treated with the indicated concentration of AD, and cell death was quantified though flow cytometry by measuring PI-positive cells. **p < 0.01, ***p < 0.001.

Arnicolide D (AD) (Fig. 1A) is a sesquiterpene lactone derived from *Centipeda minima*, a well-known traditional medicinal herb utilized for treating rhinitis, alleviating pain, and reducing swelling. In recent years, the attention on the anticancer properties of *Centipeda minima* has grown steadily [20]. While numerous natural compounds, such as brevillin A, AD, arnicolide C, chlorogenic acid, and others, have been identified in it [21], AD has emerged as the most potent in terms of anticancer activity [22]. Recent research has underscored its efficacy against various cancer types, including triple negative breast cancer (TNBC) [23], nasopharyngeal cancer (NPC) [24], melanoma [25], and colon cancer [26]. However, its specific impact on hepatocellular carcinoma (HCC) remains uncertain.

In our study, we investigated AD’s anticancer potential against HCC both in vitro and in vivo. Remarkably, we observed that AD induced ROS-dependent oncosis in HCC cells, linked to the activation of the PERK-eIF2α-ATF4-CHOP axis within the ER stress pathway. Furthermore, our findings demonstrated that both ATF4 and CHOP played critical roles in AD-induced oncosis. In summary, this study advances our understanding of AD’s anticancer properties, the underlying oncosis mechanism, and suggests the potential utility of AD as an anti-HCC agent in future therapeutic approaches.

## Results

### AD induces cell death in HCC cells

As shown in Fig. 1A, AD is a natural sesquiterpene lactone classified as a terpenoid secondary metabolite. To evaluate its potential as an anti-HCC agent, we conducted an initial MTT assay. The results, presented in Fig. 1B, C, demonstrate that AD significantly reduced the viability of two HCC cell lines, HepG2 and Huh7, in a dose- and time-dependent manner. As listed in Supplementary Table S2, the IC_50_ values for these HCC cell lines at various time points were consistently below 10 μM. While AD also affected the viability of the normal hepatocyte cell line LO2, its impact was less pronounced when compared to its effects on HCC cells (Supplementary Fig. S1 and Supplementary Table S2), indicating its lower toxicity to normal cells. To further assess AD’s inhibitory effects on HCC cells, we conducted a colony formation assay. As shown in Fig. 1D, E, even at low concentrations, AD significantly suppressed colony formation, underscoring its ability to inhibit HCC cell proliferation. In addition to the inhibitory effect on cell proliferation, the induction of cell death has been regarded as another novel strategy for the development of anticancer drugs. The evasion of cell death is a hallmark of cancer, associating with uncontrolled cell proliferation, thereby enhancing tumor formation and treatment resistance [27]. Furthermore, it has been proven that the induction of cell death can remove potential harmful cells, thereby blocking tumor growth. In light of this, we explored whether AD could induce cell death in HCC cells using PI staining coupled with flow cytometry. As shown in Fig. 1F–H, the percentage of PI^+^ cells in both HepG2 and Huh7 was significantly increased upon exposure to AD, in a dose- and time-dependent manner. These findings strongly demonstrate that AD has a remarkable ability to induce cell death of HCC cells.

### AD-induced cell death in HCC cells is oncosis

**Fig. 2:**
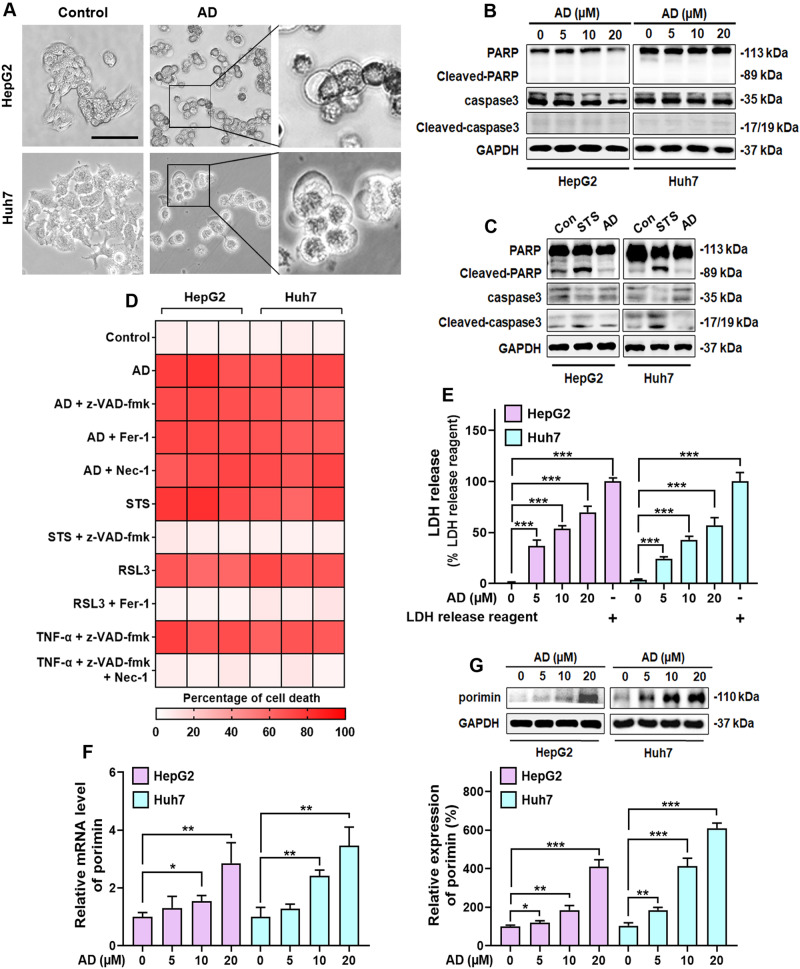
AD induced oncosis in HCC cells. **A** Morphological changes of cells exposed to 20 μM AD for 24 h. Scale bar = 50 μm. **B** No significant regulation of PARP and caspase-3 expression in cells following AD treatment. **C** Increased expression of cleaved PARP and cleaved caspase-3 in cells after treatment with the apoptotic inducer STS (2 μM) for 8 h. **D** Cell death in cells exposed to AD (20 μM) alone and in combination with various inhibitors (z-VAD-fmk, 20 μM; Fer-1, 2 μM; Nec-1: 10 μM) for 24 h. Additionally, cell death in cells separately exposed to the apoptosis inducer STS (2 μM), ferroptosis inducer RSL3 (2 μM) or the necroptosis inducer (z-VAD-fmk [20 μM]/tumor necrosis factor-α [20 ng/mL]) with or without z-VAD-fmk (20 μM), Fer-1 (2 μM) and Nec-1 (10 μM) for 24 h. **E** Lactate dehydrogenase (LDH) release in cells treated with the indicated concentration of AD for 24 h. **F** mRNA levels of porimin in cells treated with the indicated concentration of AD for 8 h. **G** Protein expression of porimin (upper panel) and histogram (lower panel) in cells treated with the indicated concentration of AD for 8 h. *p < 0.05,**p < 0.01, ***p < 0.001.

Previous studies have established that AD induces apoptosis, a major form of cell death, in other types of cancer cells [23, 24, 26]. However, our observations revealed a distinctive feature in HCC cells treated with AD. These cells exhibited swelling and the formation of membrane surface blebs, accompanied by a lack of organelles, which are not typical apoptotic features (Fig. 2A). To ascertain whether AD-induced cell death in HCC cells was apoptosis, we employed double staining with Annexin V-FITC/PI, a method distinguishing apoptotic cells. The results, as shown in Supplementary Fig. S2, indicated that the majority of cells were stained FITC^−^/PI^+^, with only a small fraction stained FITC^+^/PI^+^ or FITC^+^/PI^−^, suggesting that most cells had undergone non-apoptotic cell death. Additionally, we examined apoptotic markers such as caspase-3 and PARP under AD treatment, but neither showed significant changes (Fig. 2B). In contrast, the apoptotic inducer staurosporine (STS) significantly increased the expression of the cleaved caspase-3 and PARP (Fig. 2C). Furthermore, we conducted co-incubation of AD with z-VAD-fmk, an apoptotic inhibitor, which failed to prevent AD-induced cell death (Fig. 2D). Similarly, Ferrostatin-1 (Fer-1, a ferroptosis inhibitor) and Necrostatin-1 (Nec-1, a necroptosis inhibitor) were also ineffective in suppressing AD-induced cell death (Fig. 2D). In contrast, z-VAD-fmk inhibited cell death induced by the apoptotic inducer STS, Fer-1 inhibited cell death triggered by the ferroptosis inducer RSL3, and Nec-1 inhibited cell death induced by the necroptotic stimuli (TNF-α + z-VAD-fmk) (Fig. 2D). These findings collectively suggest that the cell death induced by AD in HCC cells does not involve apoptosis, ferroptosis or necroptosis.

Based on the observed cellular swelling and membrane blebbing, we hypothesized that AD induces oncosis, a significant non-apoptotic mode of cell death, in HCC cells. To validate this hypothesis, we measured lactate dehydrogenase (LDH) leakage to assess the integrity of the cell membrane, an indicator associated with oncosis [28]. Remarkably, AD treatment led to a significant increase in LDH release in HCC cells, as shown in Fig. 2E, suggesting a notable elevation in membrane permeability alongside morphological changes in the cells. Additionally, we investigated the regulation of porimin, a pro-oncosis receptor involved in membrane injury [29], in response to AD. The results in Fig. 2F, G demonstrated that AD treatment resulted in elevated porimin mRNA level and increased expression in HCC cells, providing further confirmation that AD-induced cell death in HCC cells is indeed oncosis.

### ER-derived ROS generation is essential for AD-induced oncosis

**Fig. 3:**
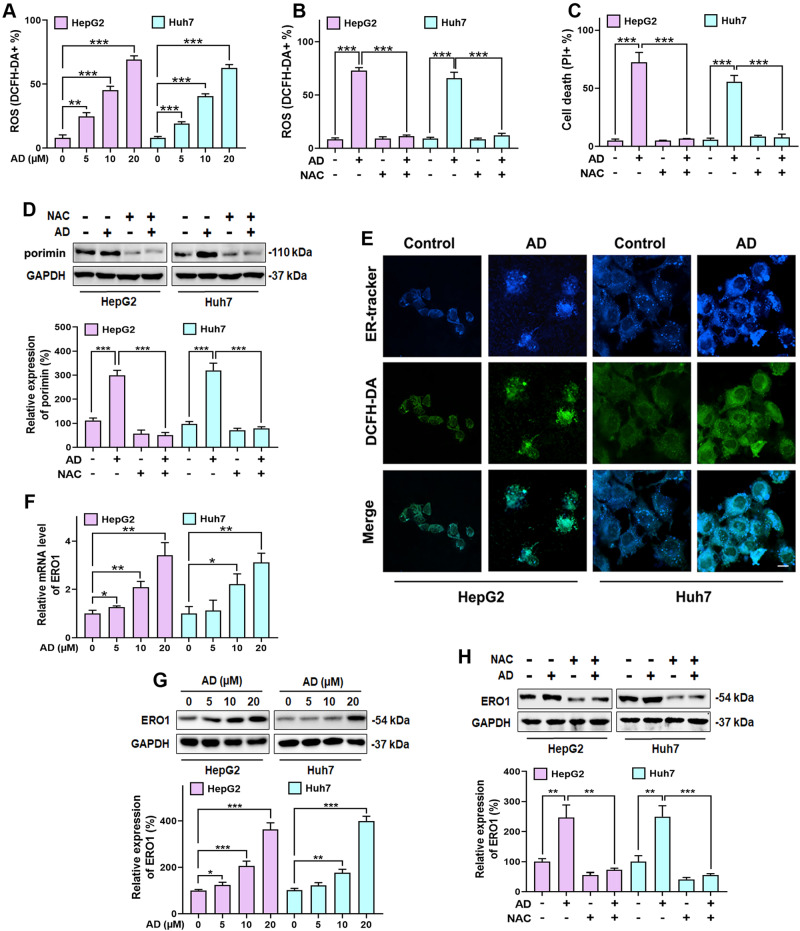
AD induced ER-derived ROS generation. **A** ROS levels in cells treated with the indicated concentration of AD for 8 h, and quantified using the DCFH-DA lipid probe by flow cytometry. **B** ROS levels and **C** cell death in cells exposed to AD (20 μM) alone and in combination with NAC (2 mM). ROS was assessed after administered for 8 h, and cell death was assessed after administered for 24 h. **D** Protein expression of porimin (upper panel) and histogram (lower panel) in cells exposed to AD (20 μM) alone and in combination with NAC (2 mM) for 8 h. **E** Images of ER-Tracker and DCFH-DA double staining in cells exposed to AD (20 μM) for 8 h. Scale bar = 25 μm. **F** mRNA levels of ERO1 in cells treated with the indicated concentration for 8 h. **G** Protein expression of ERO1 (upper panel) and histogram (lower panel) in cells treated with the indicated concentration of AD for 8 h. **H** Protein expression of ERO1 (upper panel) and histogram (lower panel) in cells exposed to AD (20 μM) alone and in combination with NAC (2 mM) for 8 h. *p < 0.05,**p < 0.01, ***p < 0.001.

The overproduction of ROS has been associated with various forms of cell deaths, including oncosis [30]. To investigate ROS generation in AD-treated HCC cells, we initially examined cellular ROS level using DCFH-DA staining combined with flow cytometry. As demonstrated in Fig. 3A, AD treatment resulted in a significant dose-dependent accumulation of ROS in HCC cells. To confirm the role of ROS in AD’s action, we utilized N-Acetyl-L-cysteine (NAC), a ROS scavenger. The co-treatment with NAC not only effectively inhibited the accumulation of ROS induced by AD (Fig. 3B), but also displayed the remarkable capability to reverse AD-induced oncosis (Fig. 3C). These findings emphasize the crucial role of ROS in inducing oncosis in HCC cells when exposed to AD. Importantly, NAC’s ability to effectively suppress the increased expression of porimin (Fig. 3D), serves as a confirmation of the ROS-dependent nature of AD-induced oncosis.

Since both mitochondria and the ER are known contributors to cellular ROS generation [31], we further investigated the intracellular distribution of ROS using double staining with DCFA-DA and either Mito-Tracker Red or ER-Tracker Blue. As shown in Fig. 3E and Supplementary Fig. S3, the green fluorescence from DCFH-DA exhibited significant overlap with the ER Tracker Blue in the cytoplasmic regions but not with the mitochondrial red fluorescence, indicating that the ER is the source of AD-induced ROS generation, rather than the mitochondria. Moreover, Mito-Tracker Red-based flow cytometry in Supplementary Fig. S4 showed only a minimal increase in mitochondrial ROS (mitoROS) following AD treatment, which was consistent with live cell imaging in Supplementary Fig. S3. To further validate the role of the ER in AD-induced ROS generation, we analyzed the regulation of ER oxidoreductin 1 (ERO1), an oxidoreductase enzyme responsible for generating ROS in the ER. The results in Fig. 3F, G demonstrated a significant increase in both ERO1 mRNA level and protein expression upon AD treatment. Additionally, NAC blocked the AD-induced increase in ERO1 expression, as shown in Fig. 3H, confirming that AD induces ER-derived ROS in HCC cells, which is essential for inducing oncosis.

### PERK-eIF2α-ATF4-CHOP axis of ER stress is involved in AD-induced oncosis

**Fig. 4:**
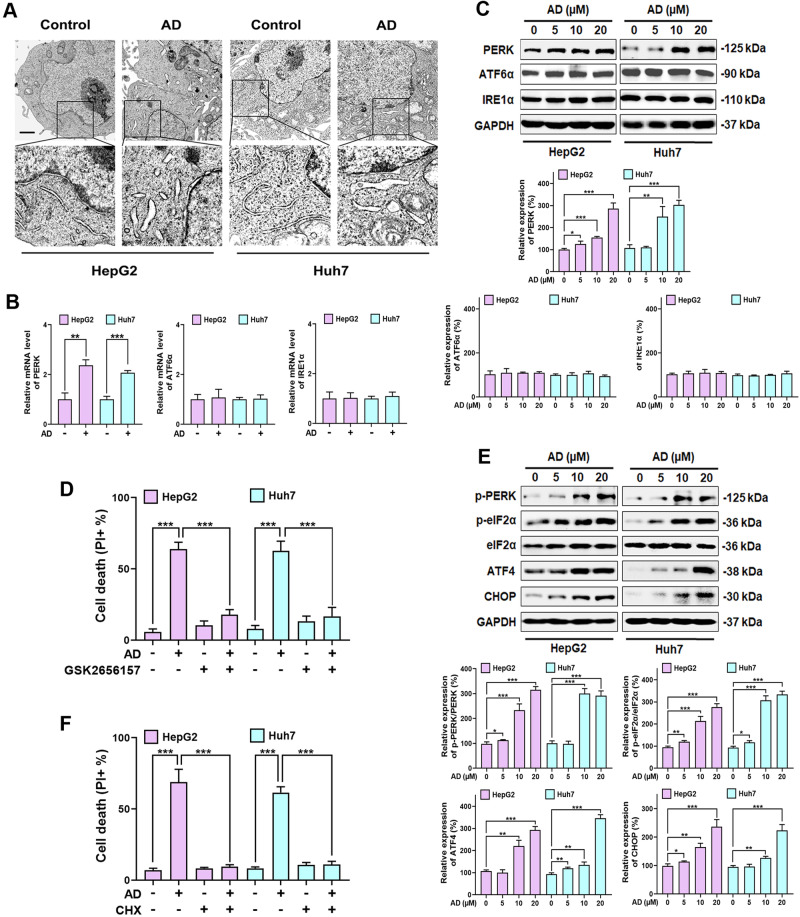
AD activated the PERK-eIF2α-ATF4-CHOP pathway during ER stress. **A** TEM images illustrating alterations in ER morphology in cells exposed to AD (20 μM) for 8 h. Scale bar = 25 μm. **B** mRNA levels of IRE1α, ATF6α and PERK in cells exposed to AD (20 μM) for 8 h. **p < 0.01. **C** Protein expression of IRE1α, ATF6α and PERK (upper panel) and histogram (lower panel) in cells treated with the indicated concentration of AD for 8 h. **D** Cell death of cells exposed to AD (20 μM) alone or in combination with GSK2656157 (2 μM) for 24 h. **E** Protein expression of the PERK-eIF2α-ATF4-CHOP pathway (upper panel) and histogram (lower panel) in cells treated with the indicated concentration of AD for 8 h. **F** Cell death of cells exposed to AD (20 μM) alone or in combination with CHX (20 μM) for 24 h. **p < 0.01.

Since AD-induced ROS production is associated with the ER, it is reasonable to assume that AD can trigger ER stress and activate Unfolded Protein Response (UPR) [32]. To investigate this, we conducted transmission electron microscopy (TEM) analysis, revealing significant alterations in ER morphology in AD-treated HCC cells, including dilation and swelling (Fig. 4A). These findings confirm that AD induces ER stress. UPR consists of three separate branches: inositol-requiring enzyme-1α (IRE1α), protein kinase R (PKR)-like endoplasmic reticulum kinase (PERK), and activating transcription factor 6α (ATF6α) [33]. To identify which signaling branch is involved in AD-induced ER stress, we examined the mRNA levels and protein expressions of these three UPR activators. Our observations showed that AD specifically increased the mRNA level and expression of PERK in HCC cells, while IRE1α and ATF6α remained relatively unchanged (Fig. 4B, C). To further confirm the role of the PERK pathway, we used GSK2656157, a specific PERK inhibitor, which effectively prevented AD-induced cell death (Fig. 4D). Additionally, the increased expression of porimin and ERO1 induced by AD was reversed (Supplementary Fig. S5), confirming the activation of the PERK branch in response to AD-induced ER stress. In the activation of the PERK pathway, PERK-mediated eIF2α phosphorylation, along with subsequent activation of ATF4 and CHOP, is one of the mechanisms of ER stress-induced cell death [34]. In this context, we investigated whether AD induced the PERK-eIF2α-ATF4-CHOP axis of ER stress in HCC cells. Our results showed that AD increased the phosphorylation of PERK and eIF2α, as well as the expression of ATF4 and CHOP (Fig. 4E), confirming the involvement of the PERK-eIF2α-ATF4-CHOP pathway in AD-induced ER stress.

Notably, PERK-mediated eIF2α phosphorylation serves as a molecular switch that determines the balance between cell survival and death by regulating protein synthesis [35]. While eIF2α phosphorylation reduces global protein synthesis, it preferentially translates downstream ATF4 [36]. However, the activation of ATF4, along with downstream CHOP, promotes protein synthesis. If protein synthesis increases before restoration, it can lead to cell death [37, 38]. Based on this, we hypothesize that AD induces oncosis in HCC cells by disrupting protein homeostasis in the ER. To confirm the effect of regulating protein synthesis by AD, we used CHX, a protein synthesis inhibitor, which was found to inhibit cell death induced by AD (Fig. 4F). Additionally, CHX reversed the activation of the PERK-eIF2α-ATF4-CHOP pathway regulated by AD (Supplementary Fig. S6), suggesting that ATF4 and CHOP may play a pivotal role in AD’s mechanisms.

### ATF4 and CHOP both regulate AD-induced oncosis

**Fig. 5:**
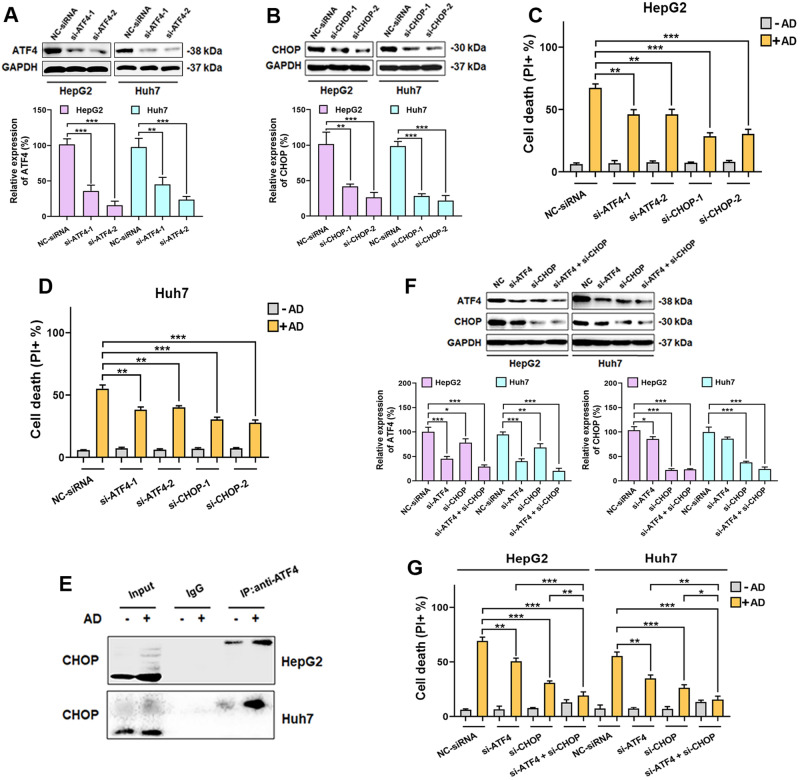
ATF4 and CHOP regulated AD-induced ROS and oncosis. **A** Expression levels of ATF4 and **B** CHOP in cells following siRNAs-mediated knockdown, separately. The lower panel shows the respective knockdown efficiency for each. **C** HepG2 and **D** Huh7 cells were exposed to AD (20 μM) after siRNAs-mediated knockdown of ATF4 or CHOP, separately. After that, cell death was measured. **E** The interaction between ATF4 and CHOP in cells exposed to AD (20 μM). **F** Simultaneous knockdown of ATF4 and CHOP by siRNAs in cells. The lower panel shows the respective knockdown efficiency for each. **G** Cell death influenced by AD (20 μM) in cells with simultaneous knockdown of ATF4 and CHOP using siRNAs. *p < 0.05, **p < 0.01, ***p < 0.001.

In subsequent experiments, we used siRNAs to individually knock down the expression of ATF4 or CHOP in HCC cells (Fig. 5A, B). We observed that knocking down either ATF4 or CHOP alone attenuated AD-induced cell death (Fig. 5C, D). Importantly, the individual knockdown of ATF4 or CHOP also mitigated the elevated levels of porimin and ERO1 induced by AD consistently (Supplementary Figs. S7 and S8). Notably, a previous study emphasized the importance of the interaction between ATF4 and CHOP in ER stress-mediated cell death [39]. To investigate whether AD influences the interaction between ATF4 and CHOP, we conducted co-immunoprecipitation (co-IP) experiments. The results in Fig. 5E indicated that the interaction between ATF4 and CHOP was strengthened under AD treatment. To further demonstrate the importance of the ATF4-CHOP interaction in AD-induced oncosis, we performed double knockdown experiments using siRNAs targeting both ATF4 and CHOP in HCC cells (Fig. 5F). Remarkably, in ATF4-CHOP double knockdown HCC cells, AD treatment had no effect on inducing cell death (Fig. 5G), confirming that both ATF4 and CHOP were essential for AD-induced oncosis.

Interestingly, bioinformatics analysis based on the TCGA database revealed higher expression levels of both ATF4 and CHOP in HCC tissues from patients (Supplementary Fig. S9). Moreover, the higher expression of both ATF4 and CHOP was significantly associated with longer survival of HCC patients (Supplementary Fig. S10). This information not only support our study but also suggests that AD treatment for HCC, mediated by the activation of ATF4 and CHOP, is a viable approach.

### AD inhibits tumor growth in xenograft-bearing nude mice

**Fig. 6:**
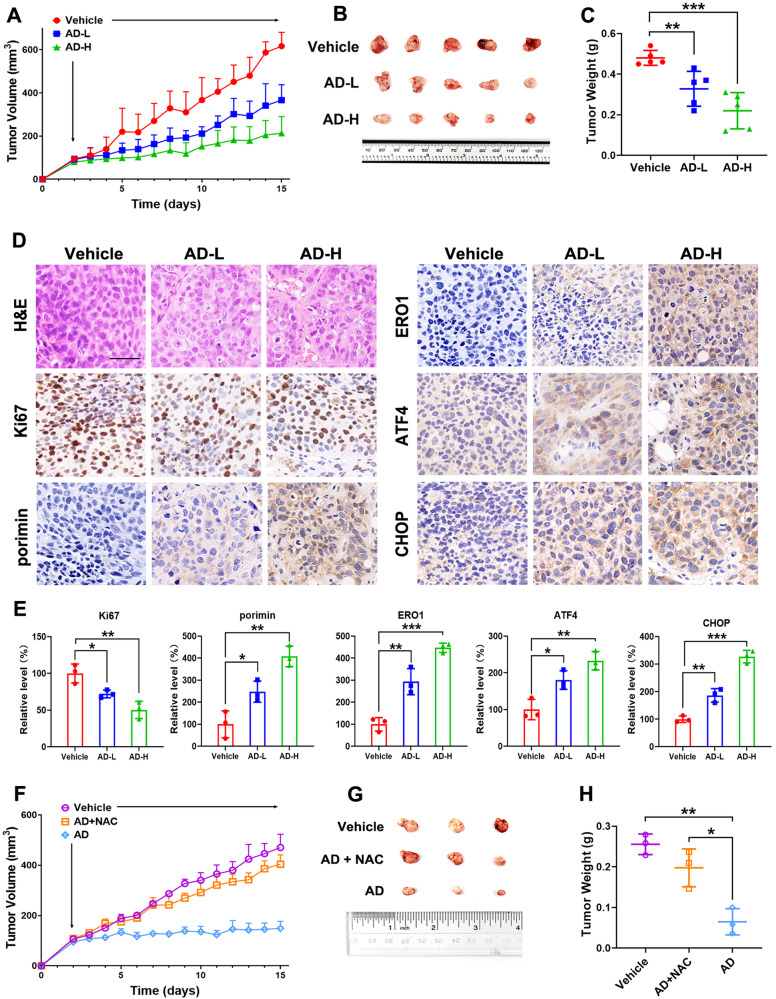
AD inhibited Huh7 xenograft tumor growth. **A**–**D** The xenograft model was established to investigate the inhibitory effect of AD on HCC tumor by subcutaneously injecting Huh7 cells into mice; the mice were randomly divided into three groups and received daily i.p. injection of either vehicle (saline), AD-L (5 mg/kg), or AD-H (20 mg/kg). **A** Tumor volumes were consistently measured throughout the experimental period. **B** Images of tumors were captured at the endpoint of experiment. **C** Tumor weights were recorded at the endpoint of experiment. **D** Xenografted tumors underwent sectioning, fixation, and staining with H&E. Additionally, immunohistochemical staining was performed for Ki67, porimin, ERO1, ATF4 and CHOP. Scale bar = 50 μm. **E** Quantitative analysis of protein expression in tumors. **F**–**H** Another xenograft model was established through the subcutaneous injection of Huh7 cells, with the primary goal of investigating the role of ROS in AD’s anti-HCC effects; the mice were randomly divided into three groups and received daily i.p. injection of either vehicle (saline), AD (20 mg/kg), or AD (20 mg/kg) + NAC (50 mg/kg). **F** Tumor volumes were monitored throughout the experiment. **G** Images of tumors were captured, and **H** Tumor weights were measured. *p < 0.05, **p < 0.01, ***p < 0.001.

To evaluate the effectiveness of AD in inhibiting tumor growth in vivo, we established a nude mice xenograft model by subcutaneously injecting Huh7 cells. After tumor inoculation, the mice were given daily doses of either a low dose (5 mg/kg) or a high dose (20 mg/kg) of AD for 14 consecutive days. At the end of this 14-day treatment period, the mice were euthanized, and tumor burden was compared between the vehicle (saline treatment) and AD-treated groups. The results presented in Fig. 6A–C clearly showed a significant increase in tumor volumes and masses in the vehicle group. In contrast, in the mice treated with AD, especially at the high dose, the tumor volumes and masses were notably smaller compared to the vehicle group. Importantly, the body weight of the mice remained unchanged in both AD-treated groups, as shown in Supplementary Fig. S11. Furthermore, histological analysis of tumor sections in Fig. 6D revealed densely packed cells in the tumors of the vehicle group. In contrast, the tumors in the AD-treated groups, especially in the high-dose group, displayed the presence of vacuoles. This observation was supported by a significant reduction in Ki67 expression, confirming the potent anti-HCC activity of AD in vivo (Fig. 6D). Importantly, the expression levels of porimin, ERO1, ATF4 and CHOP were significantly upregulated (Fig. 6D, E), serving as robust indicators of ER stress-mediated oncosis triggered by AD treatment. Interesting, the introduction of NAC effectively reversed the tumor suppressive impact of AD (Fig. 6F–H), emphasizing the ROS-dependent nature of its anti-HCC efficacy.

## Discussion

HCC is the most common primary liver cancer, accounting for 75–85% of cases. In 2020, it ranked as the most frequently diagnosed cancer and the third leading cause of cancer-related deaths worldwide [40]. Surgical treatments like liver transplantation and resection work for early-stage HCC, but most patients are diagnosed at advanced stages, ruling out surgery. Therefore, chemotherapy, typically using agents such as 5-fluorouracil, cisplatin, and adriamycin, remains the predominant choice, despite their side effects and limited effectiveness [41]. Sorafenib gained FDA approval as a systemic therapy for advanced HCC in 2016, increasing median survival time with manageable side effects [42]. However, sorafenib resistance remains a major challenge in clinical HCC treatment [43]. Hence, there’s urgent need to explore novel strategies and identify new drugs for HCC treatment.

In this study, we present compelling evidence of the therapeutic potential of AD, a natural sesquiterpene lactone compound derived from *Centipeda minima*, against HCC both in vitro and in vivo. Remarkably, we found that AD induces a non-apoptotic form of cell death in HCC cells.

While apoptosis has traditionally been a central focus of cancer therapy, its evasion by cancer cells often leads to treatment resistance and recurrence [44]. Consequently, researchers have shifted their attention to therapeutic agents capable of inducing non-apoptotic cell death. Over the past few decades, oncosis, a non-apoptotic cell death mechanism, has garnered attention as a potential strategy for anticancer drug development. In our study, HCC cells treated with AD exhibited distinctive features, including cellular swelling and membrane surface blebs with organelle-free regions, characteristics not typical of apoptosis. Importantly, experiments with various pharmacological inhibitors specific to different cell death pathways demonstrated that AD-induced cell death was unresponsive to these inhibitors. Furthermore, AD was found to induce membrane damage and elevate porimin levels in HCC cells, confirming that AD-induced cell death is oncosis.

It is important to recognize that a single compound can induce different forms of cell death, influenced by factors such as cell type, drug concentration, and duration of exposure. For instance, paclitaxel, acknowledged for its primary induction of apoptosis [45], has been observed to trigger ferroptosis [46], necroptosis [47], oncosis [48], and paraptosis [49] in different cancer cells. Despite previous research indicating that AD induces apoptosis in various cancer cells, our current findings reveal that in HCC cells, AD prompts oncosis. This discrepancy is understandable and likely attributed to the diversity of tumor types under consideration [50].

ROS have been associated with various cell death mechanisms and can interplay with different types of cell death as a critical regulator [51]. In our subsequent investigations, we explored the role of ROS in AD-induced oncosis. AD was observed to increase ROS level in HCC cells. Significantly, the inhibition of ROS by NAC, a ROS scavenger, effectively blocked AD-induced cell death, confirming the ROS-dependent nature of AD-induced oncosis. Through double staining and examination of ERO1 expression, we concluded that AD-induced ROS is primarily derived from the ER.

ROS production within the ER can lead to ER stress, which triggers the UPR [52]. In our study, we identified the activation of the PERK-eIF2α-ATF4-CHOP pathway as a key component of AD-induced ER stress. Notably, the URP initially acts as an adaptive mechanism for cell survival; however, prolonged ER stress can lead to cell death [9]. Within this intricate cellular response, PERK-mediated eIF2α phosphorylation and subsequent ATF4 activation play crucial roles [53]. While eIF2α phosphorylation can reduce global protein synthesis, it selectively promotes the translation of ATF4. The paradox here is that ATF4, along with downstream CHOP, can promote protein synthesis and, if unchecked, lead to cell death [39]. Our experiments showed that CHX, a protein synthesis inhibitor, effectively prevented cell death induced by AD, suggesting the importance of protein synthesis and promoting us to focus on ATF4 and CHOP in AD’s mechanism of action. Further analysis revealed that the simultaneous knockdown of both ATF4 and CHOP fully abolished the effects of AD, emphasizing their essential roles. Subsequent animal experiments provided further validation of AD’s inhibitory effect on HCC tumor growth. Notably, the in vivo anti-HCC activity of AD was confirmed to be intricately linked to the induction of ER stress-mediated oncosis, a process that is ROS-dependent.

In conclusion, this study provides valuable insights into the potential therapeutic effects of AD against HCC. AD demonstrates anti-HCC activity by inducing ER-mediated and ROS-dependent oncosis. Moreover, both ATF4 and CHOP in the PERK pathway play crucial roles for AD-induced oncosis. This study highlights AD’s potential as a therapeutic option for HCC, offering a novel avenue for addressing this challenging malignancy.

## Materials and methods

### Reagents

Arnicolide D (C_19_H_24_O_5_, CAS34532-68-8) was purchased from Jiangsu Yongjian Pharmaceutical Co., Ltd (Jiangsu, China). Chemical reagents, including N-AcetylL-cysteine (NAC), propidium iodide (PI), z-VAD-fmk, ferrostatin-1, necrostatin-1, cycloheximide (CHX), and GSK2656157, were obtained from MedChemExpress (Shanghai, China). Dulbecco’s Modified Eagle’s Medium, fetal bovine serum, and Lipofectamine™ 3000 were sourced from Thermo Fisher (MA, USA).

### Cell lines and culture

HepG2 and Huh7 cell lines were obtained from American Type Culture Collection (ATCC). The cells were cultured in DMEM supplemented with 10% FBS, whitin a humidified environment at 37 °C, with a composition of 5% CO_2_ and 95% air. The culture medium was refreshed every three days, and cell passaging was carried out using 0.05% trypsin/EDTA.

### MTT assay

The effect of AD on the proliferation and viability of HCC cells was assessed using the 3-(4,5-dimethylthiazol-2-yl)-2,5-diphenyltetrazolium bromide (MTT) uptake method. In brief, cells were initially seeded at a density of 5 × 10^3^ cells/well in 96-well plates 24 h before AD treatment. The evaluation of AD’s influence on HCC cell viability was conducted at various concentrations and time points. Independent experiments were performed in triplicate. The half-maximal inhibitory concentration (IC_50_) values were determined using Graphpad Prism 5.

### Colony formation assay

HCC cells were seeded in 6-well plates at a density of 2000 cells/well. They were subjected to treatment with various concentrations of AD for a period of 12–16 days, allowing individual cells to develop distinct visible colonies. Subsequently, the cells were fixed with anhydrous methanol for 5 min, then dried and stained with 0.1% crystal violet solution for 10 min at room temperature. After rinsing, the plates were air-dried, and digital images were captured. Independent experiments were performed in triplicate, and colony counts were quantified using ImageJ software (Version 1.4.3.67, NIH, Bethesda, MD, USA).

### Cell death quantification

All cell death experiments were conducted in ~70–80% confluent wells of 6-well plates. Cells were treated with AD as indicated. To quantify cell death, 1 μg/ml of PI was introduced into the culture media, and the cells were subsequently examined using flow cytometry to identify PI-positive cells. Independent experiments were performed in triplicate.

### Measurement of cellular ROS

Cells were treated as indicated and then incubated for 1 h with 20 μM of 2′,7′-dichlorodihydrofluorescein diacetate (DCFH-DA). Afterward, any excess DCFH-DA was removed by washing the cells twice with PBS. The labeled cells were trypsinized and suspended in PBS containing 5% FBS. The oxidation of H2DCFDA to the highly fluorescent 2′,7′-dichlorofluorescein (DCF) was measured in proportion to ROS generation and analyzed using a flow cytometer. Independent experiments were performed in triplicate.

### Real time PCR analysis

RNA was extracted from HCC cells using TRIzol reagent (Invitrogen), and subsequently, cDNAs were prepared via reverse transcription. Quantitative polymerase chain reaction (PCR) was carried out utilizing the Quantitect SYBR Green PCR Master Mix (Qiagen, Valencia, CA). Each reaction contained 1 μL cDNA in a final volume of 10 μL and the following primers at a final concentration of 1000 nM. Amplification of the cDNAs was conducted on the LightCycler 2000 instrument (Roche, Indianapolis, IN). The cycling conditions involved an intial denaturation step for 15 min at 95 °C, followed by 40 cycles of denaturation (95 °C for 15 s), annealing (59 °C for 20 s), and extension (72 °C for 15 s). After amplification, a melting curve analysis was performed with denaturation at 95 °C for 5 s, followed by continuous fluorescence measurement from 70 °C to 95 °C at a rate of 0.1 °C/s. Primer information is shown in Supplementary Table S1. Independent experiments were performed in triplicate.

### Western blot analysis

Proteins were extracted from HCC cells with RIPA lysis buffer, followed by centrifugation at 13,500 rpm for 15 min at 4 °C. Protein concentration was measured using Pierce (R) BCA Protein Assay Kit, and equal amount of protein was separated on 10% SDS-PAGE and transferred to PVDF membranes. After blocking (5% skim milk powder in TBS-Tween 20) for 1 h at room temperature, the membrane was then incubated with the respective primary antibody overnight at 4 °C. Afterward, the membrane was incubated with secondary antibody for 1 h at room temperature. All antibodies were diluted in TBS-Tween 20 containing 5% dry milk. The immune-reactive proteins were detected by enhanced chemiluminescence (ECL) using X-ray film and ECL reagent. The primary antibodies used were the following: porimin (Santa Cruz, 377189), ERO1 (Santa Cruz, 365526), PERK (Santa Cruz, 377400), p-PERK (Beyotime, AF5902), ATF6α (Santa Cruz, 22799), IRE1α (CST, 3294), p-eIF2α (CST, 9721), eIF2α (CST, 9722), ATF4 (Santa Cruz, 390063), CHOP (Santa Cruz, 7351), and GAPDH (Santa Cruz, 365062). HRP-goat anti-rabbit secondary antibody (Invitrogen, 32460) and HRP-goat anti-mouse secondary antibody (Invitrogen, 31430) were used.

### siRNA transfection

The siRNA sequences were provided by GenePharma (Shanghai, China). The sequences for the siRNAs targeting CHOP were 5’-GCGCAUGAAGGAGAAAGAATT-3’ and 5’-GGUCCUGUCUUCAGAUGAATT-3’. The sequences for the siRNAs targeting ATF4 were 5’-GGUGAACCCAAUUGGCCAUTT-3’ and 5’-CCAAAUAGGAGCCUCCCAUTT-3’. Transfection was carried out using Lipofectamine 3000 reagent and Opti-MEM medium, following the manufacturer’s instructions. After 72 h of transfection, the infection efficiency was assessed through western blotting assay.

### Tumor xenograft study

Male BALB/c nude mice, 6 weeks old, were procured from ZhuHai Bestest Biotechnology Co., Ltd. These mice were housed under standard conditions at room temperature of 23 ± 2 °C, with a 12 h light-dark cycle. They had ad libitum access to food and water. All animal experiments were conducted at The Hong Kong Polytechnic University in accordance with the approved protocol by the Animal Subjects Ethics Sub-committee. For the xenograft model, Huh7 cells (5 × 10^6^ suspended in 0.1 mL DMEM medium without FBS or penicillin/streptomycin) were subcutaneously inoculated into the right flank of each mouse, and tumor growth was consistently monitored. Once the average tumor volumes reached 100 mm^3^, the mice were randomly divided into three groups, each consisting of 5 mice: (i) Vehicle group, normally fed, receiving daily i.p. saline; (ii) AD-low group, normally fed, receiving daily i.p. AD (5 mg/kg/day) dissolved in 0.9% sodium chloride solution; (iii) AD-high group, normally fed, receiving daily i.p. AD (20 mg/kg/day) dissolved in 0.9% sodium chloride solution.

To evaluate the role of ROS induction in AD’s mechanism of action, we also conducted another xenograft mouse model using Huh7 cells. In this model, the mice were divided into three groups, each consisting of 3 mice: (i) Vehicle group, normally fed, receiving daily i.p. saline; (ii) AD group, normally fed, receiving daily i.p. AD (20 mg/kg/day) dissolved in 0.9% sodium chloride solution; (iii) AD + NAC group, normally fed, receiving daily i.p. AD (20 mg/kg/day) and NAC (50 mg/kg/day) dissolved in 0.9% sodium chloride solution.

Mice body weights and tumor sizes were measured daily, and tumor volumes were calculated using the formula volume = (length × width^2^)/2. All mice were humanely euthanized by CO_2_ inhalation on the 14th day post-treatment, and their tumors were harvested, weighed, stored and fixed for further analysis.

### Statistical analysis

The data were shown as mean ± standard errors with three independent experiments. Statistical analysis was conducted through one-way analysis of variance (ANOVA) comparing the samples with their respective control using GraphPad Prism 8.0 software. Data are taken as significance when *p* < 0.05.

### Supplementary information

Supplementary data

The original western blots

The reproducibility checklist

## Data Availability

The data that support the findings of this study are available on request from the corresponding author.
