# Supplementary material for: Arnicolide D induces endoplasmic reticulum stress-mediated oncosis via ATF4 and CHOP in hepatocellular carcinoma cells
Source: Cell Death Discov. 2024 Mar 12;10:134. doi: 10.1038/s41420-024-01911-w (PMC10933425; doi:10.1038/s41420-024-01911-w)
Supplement: Supplementary file 1 — Supplementary data [file 41420_2024_1911_MOESM1_ESM.pdf]

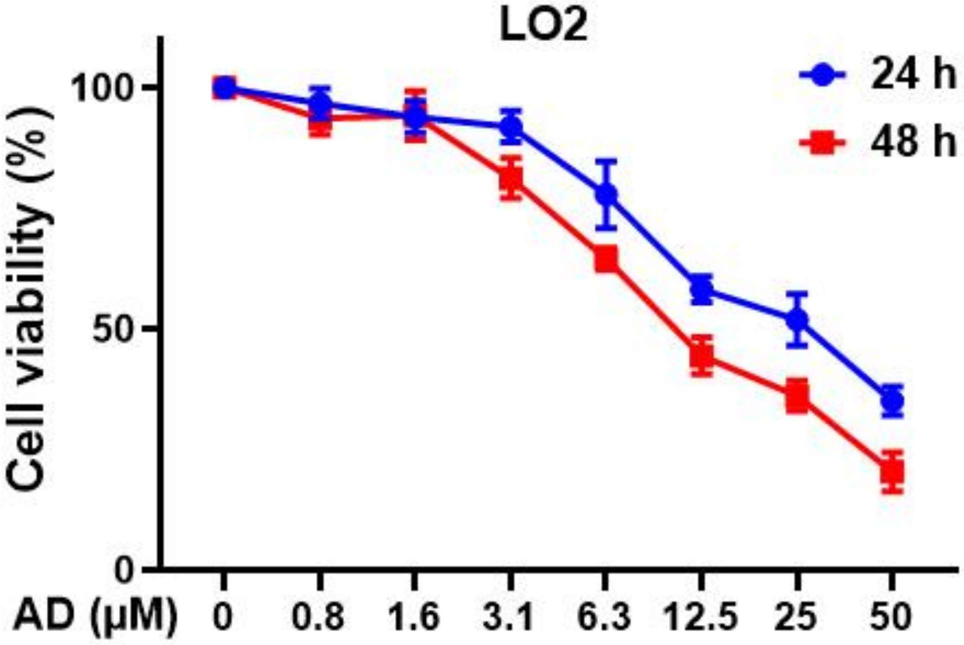

Supplementary Figure 1. MTT assay of LO2 cell line treated with increasing concentration of AD in a time course (24 h and 48 h).

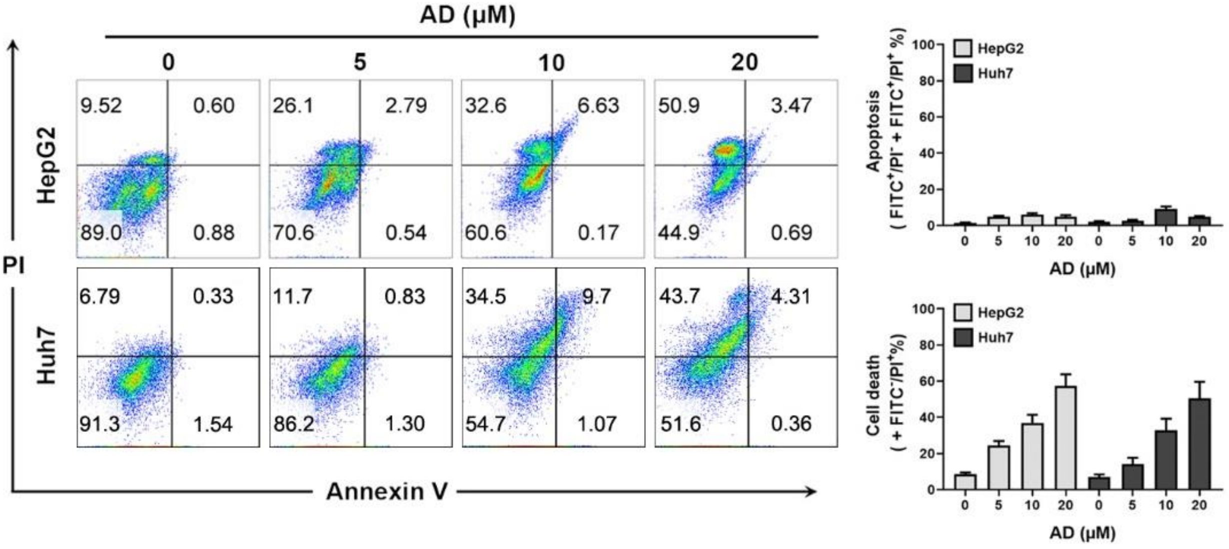

Supplementary Figure 2. Annexin V-FITC/PI staining analysis in HepG2 and Huh7 cells treated with the indicated concentration of AD for 24 h.

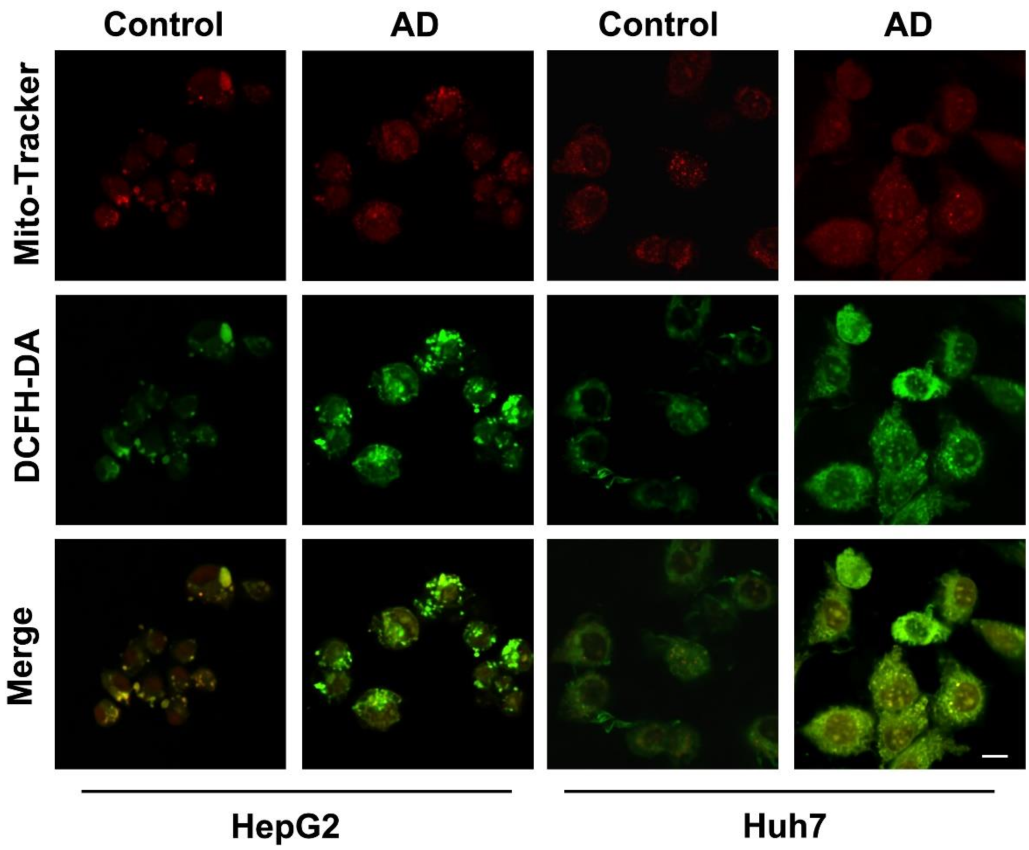

Supplementary Figure 3. Images of Mito-Tracker and DCFH-DA double staining HepG2 and Huh7 cells treated with 20 μM of AD for 8 h. Scale bar=25 μm..

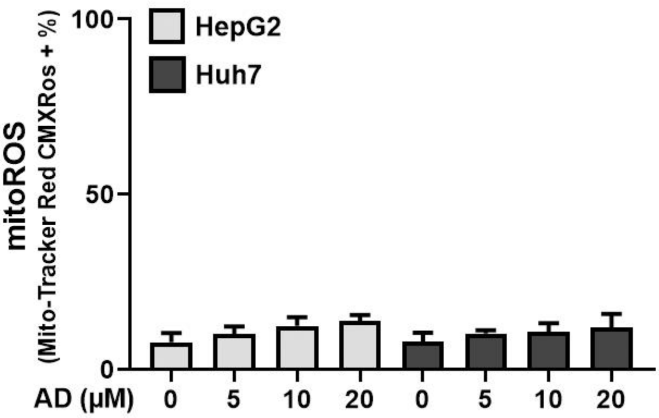

Supplementary Figure 4. The mitoROS levels in HepG2 and Huh7 cells treated with the indicated concentration of AD for 8 h, quantified using Mito-Tracker Red based-flow cytometry.

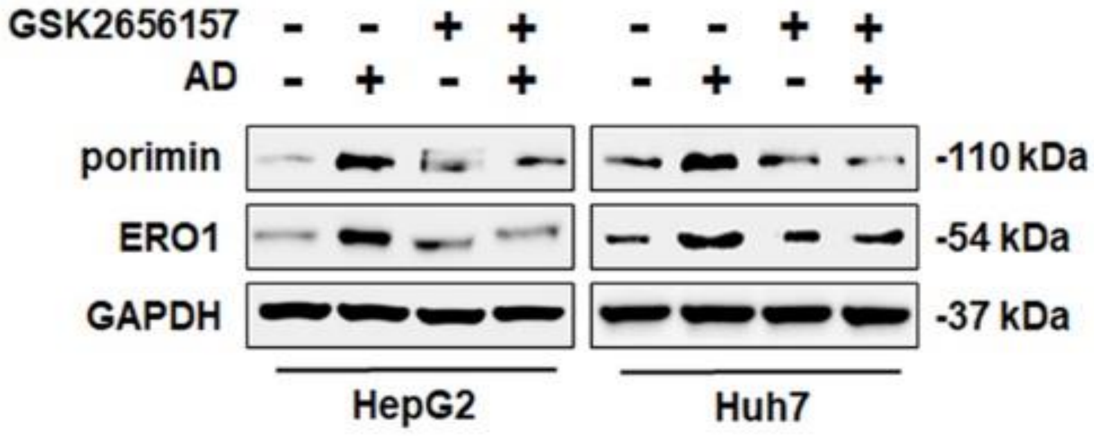

Supplementary Figure 5. Protein expression of porimin and ERO1 in cells exposed to AD (20 μM) alone or in combination with GSK2656157 (2 μM) for 8 h.

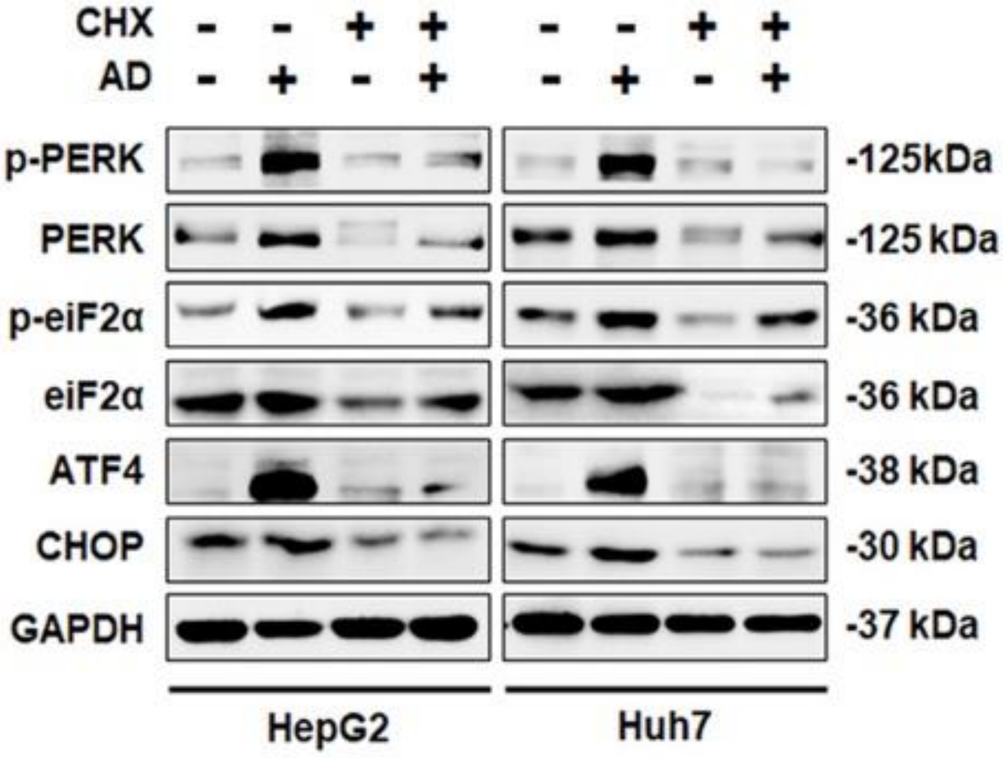

Supplementary Figure 6. Protein expression of the PERK-eIF2α-ATF4-CHOP pathway in cells exposed to AD (20 μM) alone or in combination with CHX (20 μM) for 8 h.

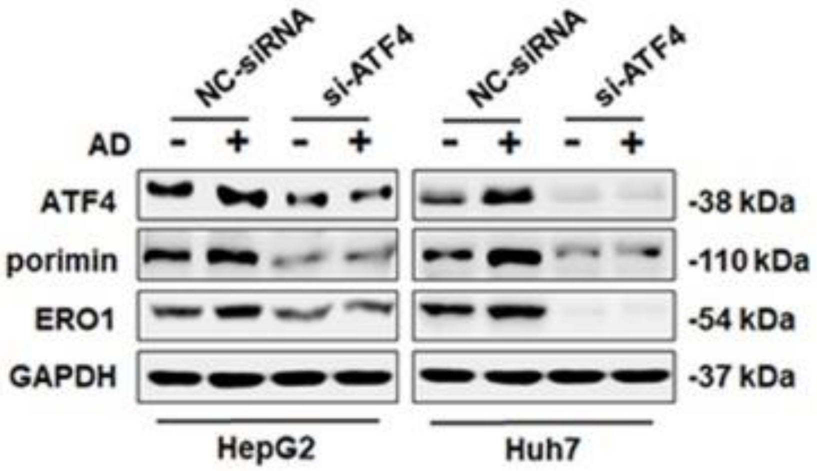

Supplementary Figure 7. Protein expression of porimin and ERO1 influenced by AD (20 μM) in cells with knockdown of ATF4.

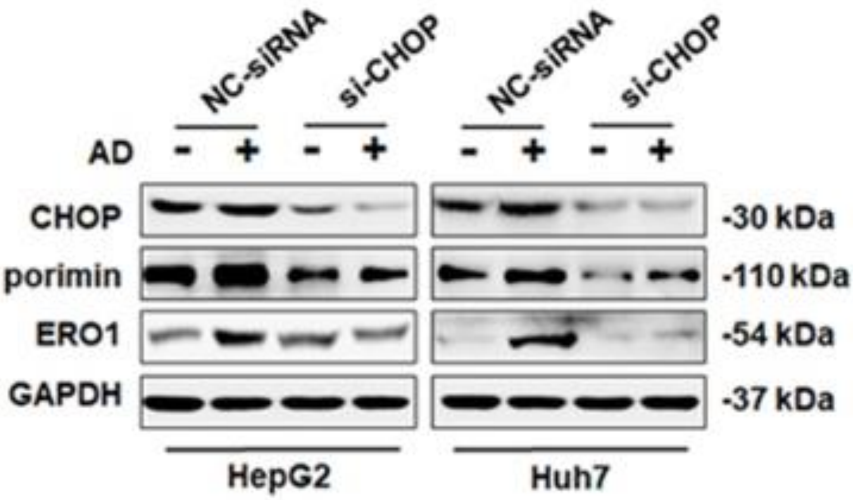

Supplementary Figure 8. Protein expression of porimin and ERO1 influenced by AD (20 μM) in cells with knockdown of CHOP.

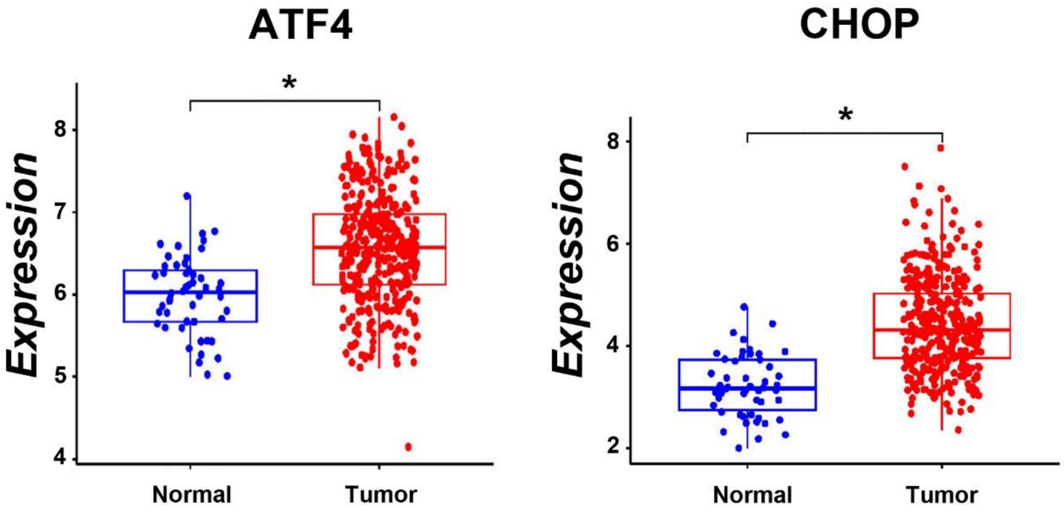

Supplementary Figure 9. Expression of ATF4 or CHOP in normal and tumor tissues was detected in the TCGA database. *$p < 0.05$.

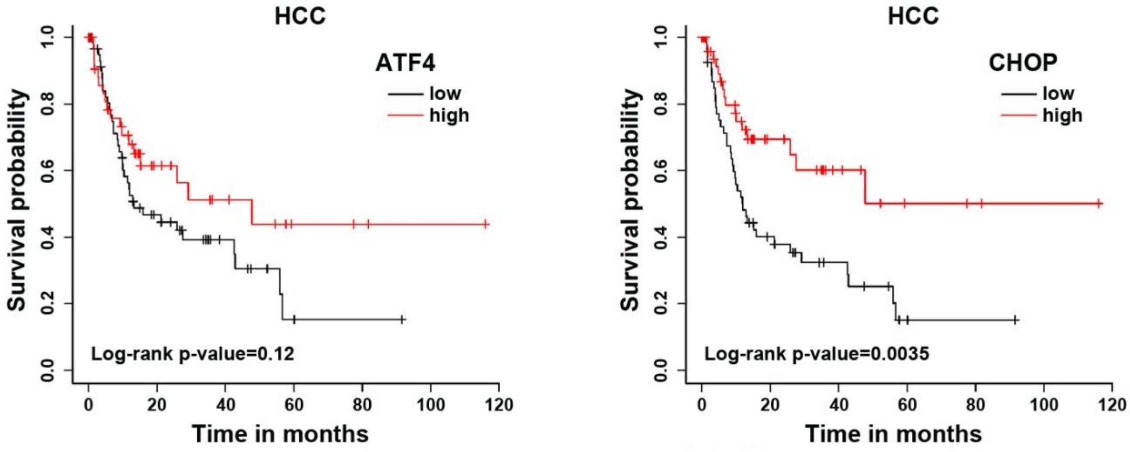

Supplementary Figure 10. Survival analysis comparing high to low expression of ATF4 or CHOP in HCC.

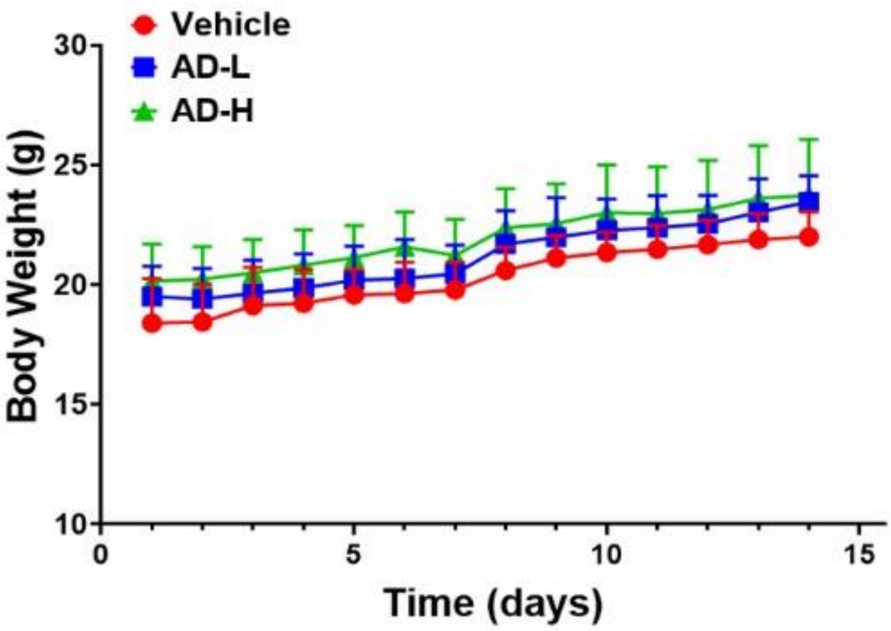

Supplementary Figure 11. The body weights of the mice were monitored throughout the experimental period.

Supplementary Table 1. The sequences of primers used for RT-PCR

mRNA	Sequence(5'→3')	
porimin	Forward primer:	AGCAGCACCGTGAAACCATT
	Reverse primer:	GGTTGTGGTCATCATGGGTG
ERO1	Forward primer:	AAGAGGCCGTGTCCTTTCTG
	Reverse primer:	CCACCAGCAGATCCAATCATC
IRE1α	Forward primer:	CGGCCTCGGGATTTTTGGA
	Reverse primer:	AGAAAGGCAGGCTCTTCCAC
ATF6α	Forward primer:	ACCCGTATTCTTCAGGGTGC
	Reverse primer:	CACTCCCTGAGTTCCTGCTG
PERK	Forward primer:	GGAGCAGGGAAGAAAAGGTCA
	Reverse primer:	TGGATGACACCAAGGAACCG

Supplementary Table 2. The IC_{50} (μM) values of AD on three cell lines

Cell lines	24 h	48 h
HepG2	8.12	4.38
Huh7	9.05	8.31
LO2	21.43	9.94
