# Supplementary material for: Arnicolide D induces endoplasmic reticulum stress-mediated oncosis via ATF4 and CHOP in hepatocellular carcinoma cells
Source: Cell Death Discov. 2024 Mar 12;10:134. doi: 10.1038/s41420-024-01911-w (PMC10933425; doi:10.1038/s41420-024-01911-w)
Supplement: Supplementary file 2 — The original western blots [file 41420_2024_1911_MOESM2_ESM.pdf]

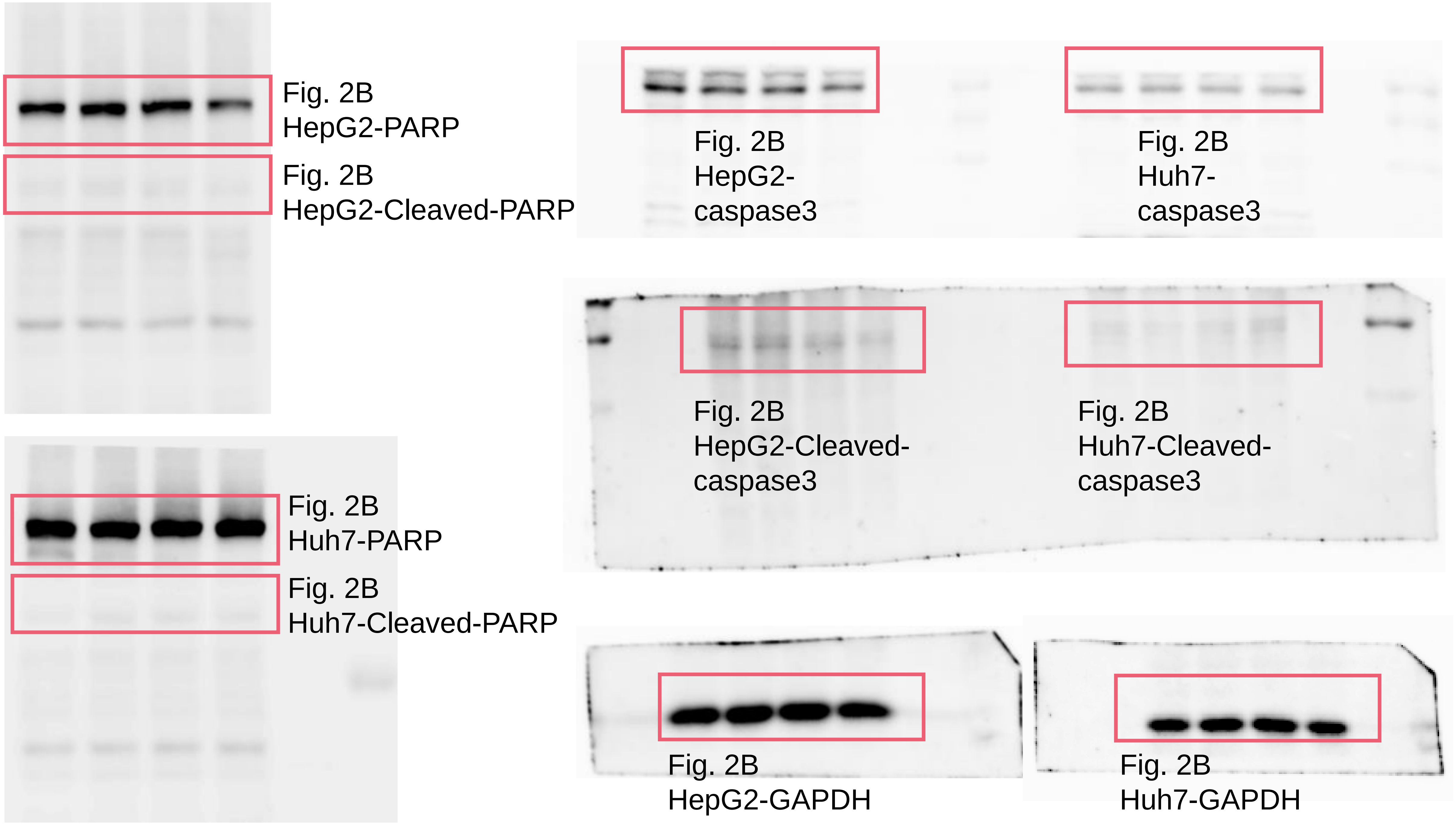
Fig. 2B HepG2-PARP
Fig. 2B HepG2-Cleaved-PARP
Fig. 2B Huh7-PARP
Fig. 2B Huh7-Cleaved-PARP
Fig. 2B HepG2-caspase3
Fig. 2B Huh7-caspase3
Fig. 2B HepG2-Cleaved-caspase3
Fig. 2B Huh7-Cleaved-caspase3
Fig. 2B HepG2-GAPDH
Fig. 2B Huh7-GAPDH

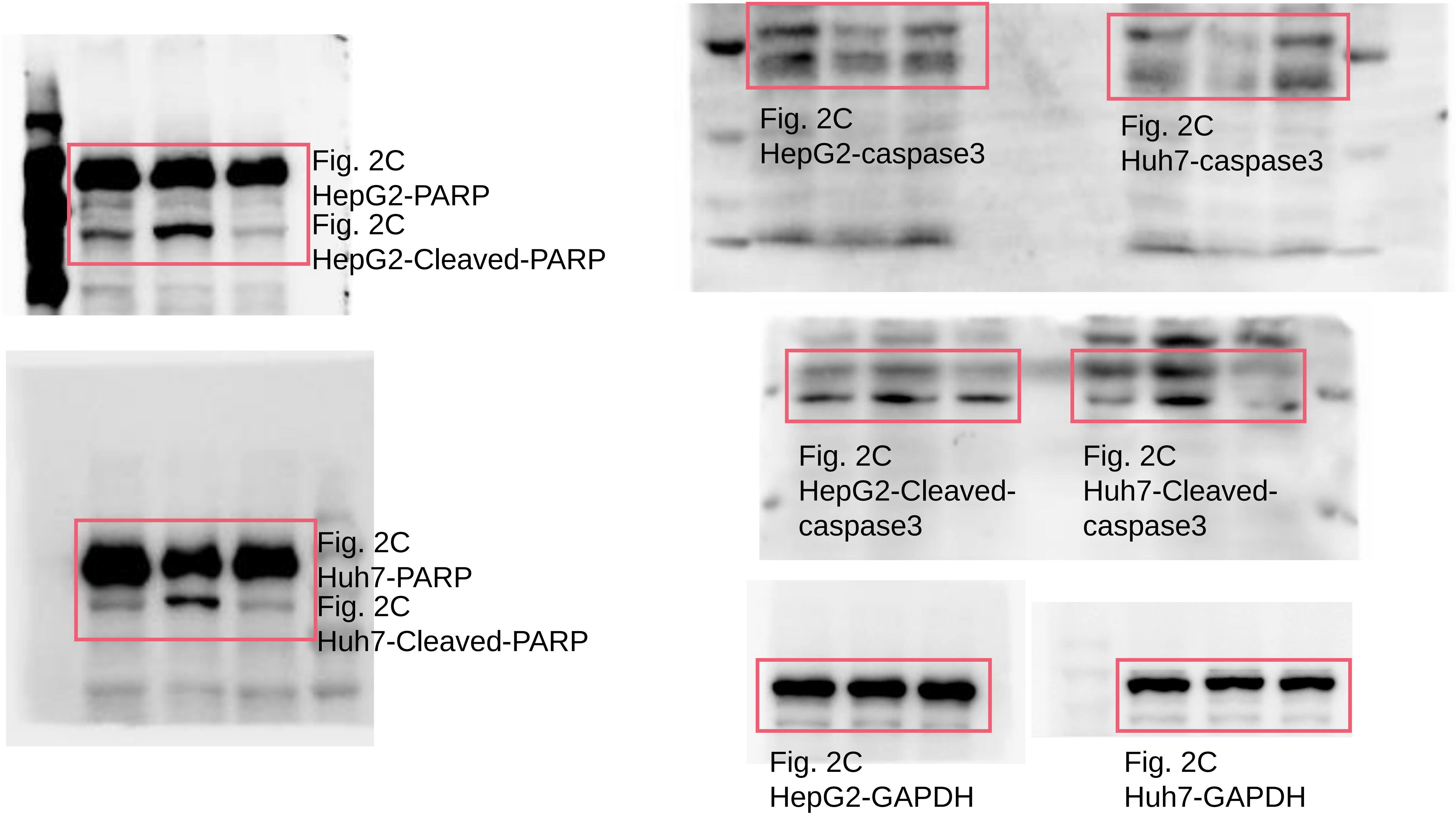

Fig. 2C
HepG2-PARP
Fig. 2C
HepG2-Cleaved-PARP
Fig. 2C
Huh7-PARP
Fig. 2C
Huh7-Cleaved-PARP
Fig. 2C
HepG2-caspase3
Fig. 2C
Huh7-caspase3
Fig. 2C
HepG2-Cleaved-caspase3
Fig. 2C
Huh7-Cleaved-caspase3
Fig. 2C
HepG2-GAPDH
Fig. 2C
Huh7-GAPDH

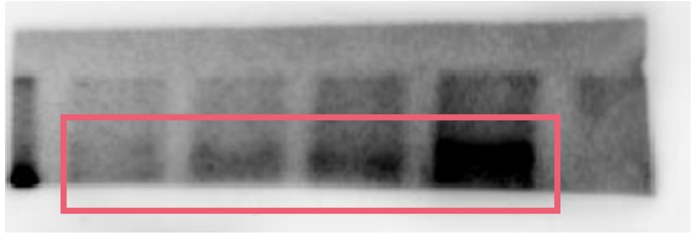

Fig. 2G
HepG2-porimin

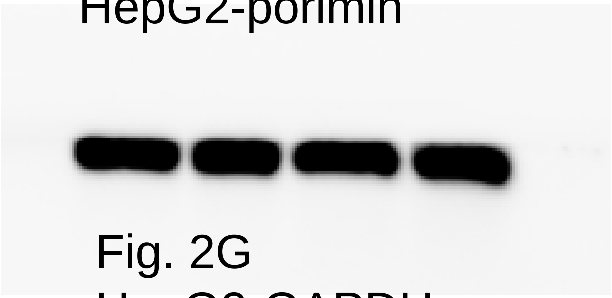

Fig. 2G
HepG2-GAPDH

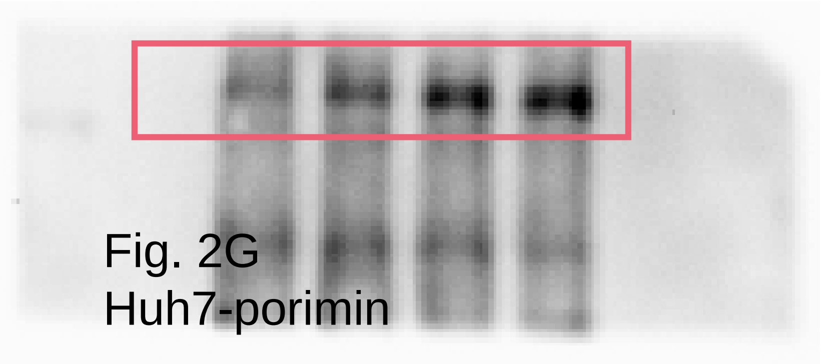

Fig. 2G
Huh7-porimin

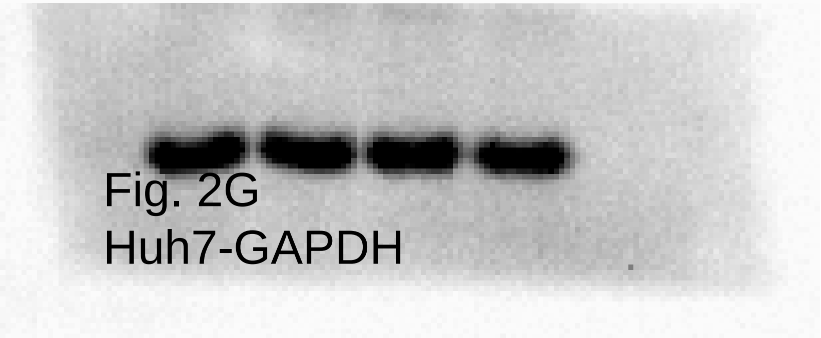

Fig. 2G
Huh7-GAPDH

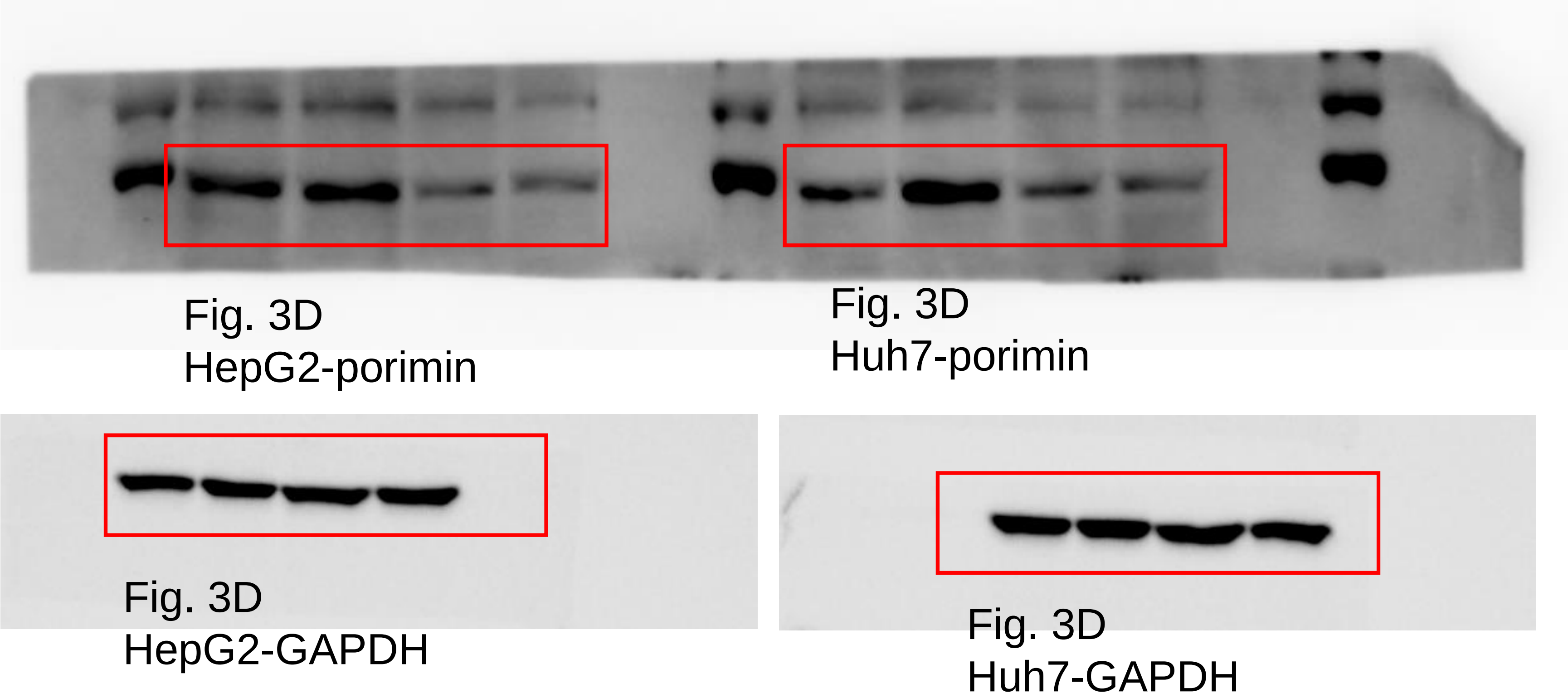
Fig. 3D
HepG2-porimin
Fig. 3D
Huh7-porimin
Fig. 3D
HepG2-GAPDH
Fig. 3D
Huh7-GAPDH

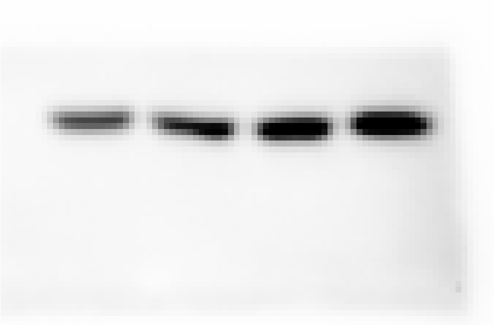

Fig. 3G
HepG2-ERO1

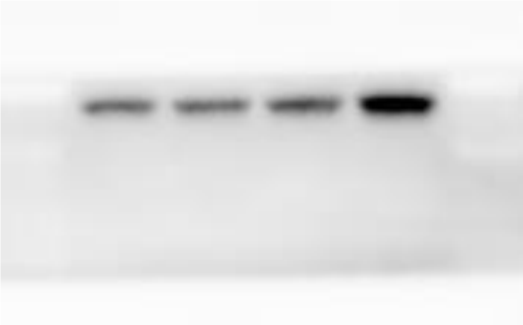

Fig. 3G
Huh7-ERO1

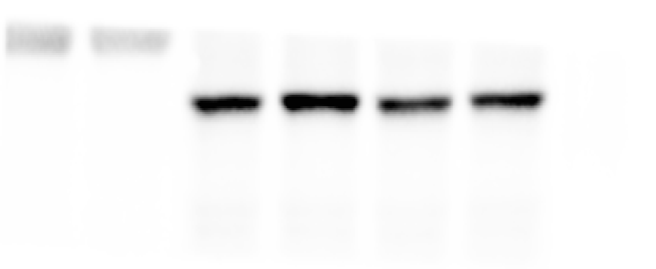

Fig. 3H
HepG2-ERO1

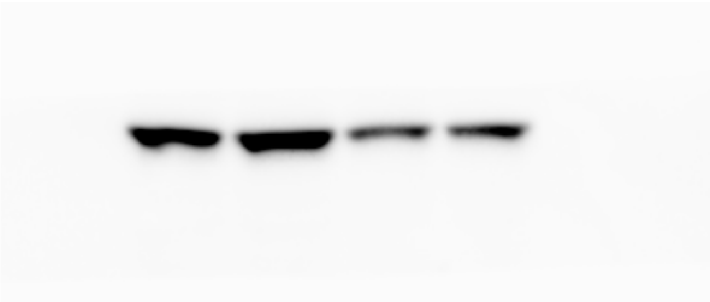

Fig. 3H
Huh7-ERO1

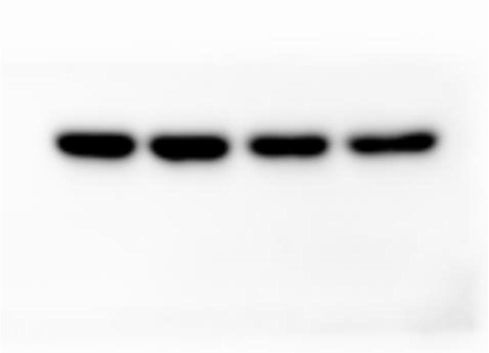

Fig. 3G
HepG2-GAPDH

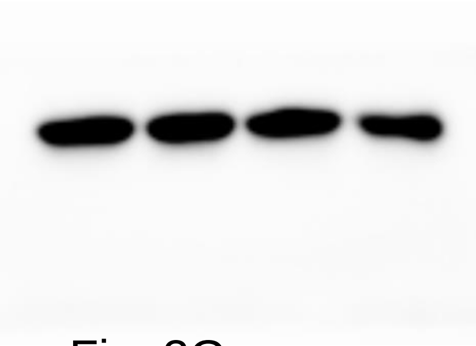

Fig. 3G
Huh7-GAPDH

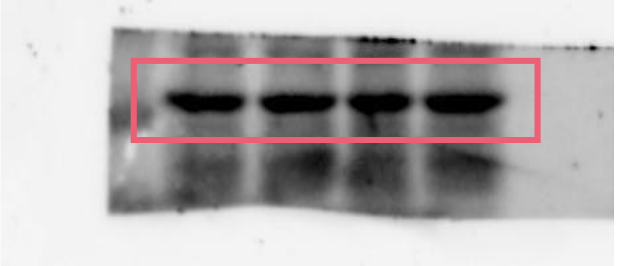

Fig. 3H
HepG2-GAPDH

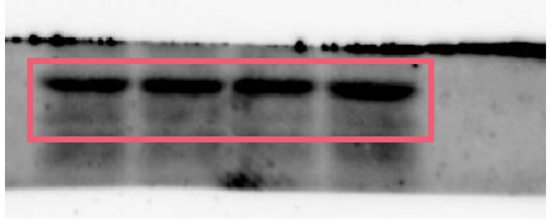

Fig. 3H
Huh7-GAPDH

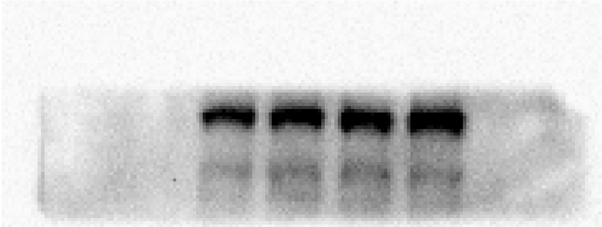

Fig. 4C
HepG2-PERK

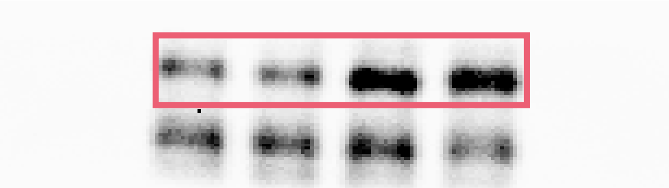

Fig. 4C
Huh7-PERK

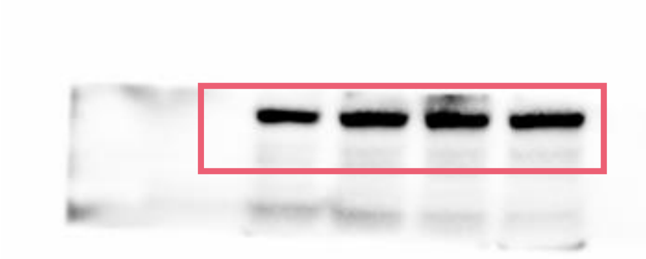

Fig. 4C
HepG2-ATF-6α

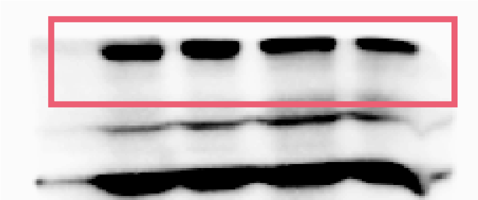

Fig. 4C
Huh7-ATF-6α

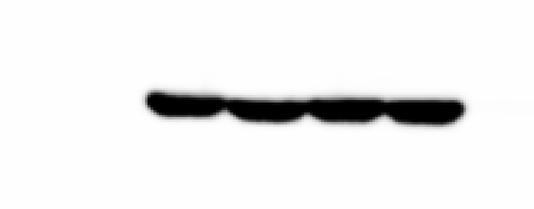

Fig. 4C
HepG2-GAPDH

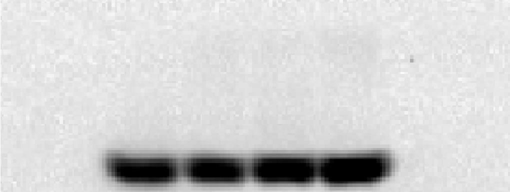

Fig. 4C
Huh7-GAPDH

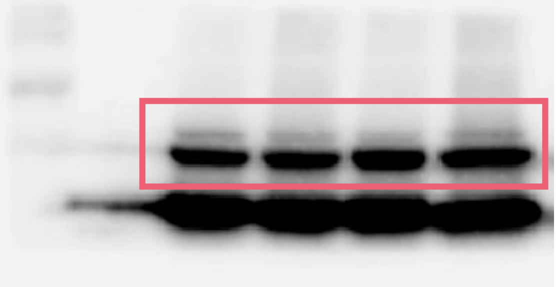

Fig. 4C
HepG2-IRE 1α

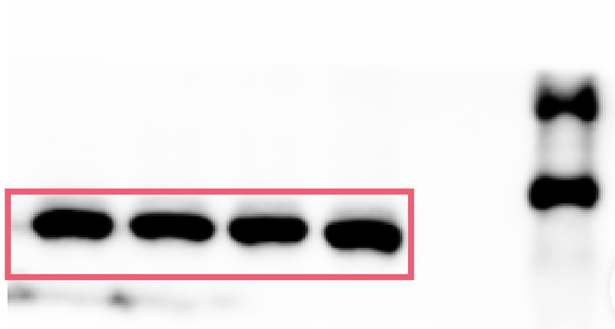

Fig. 4C
Huh7-IRE 1α

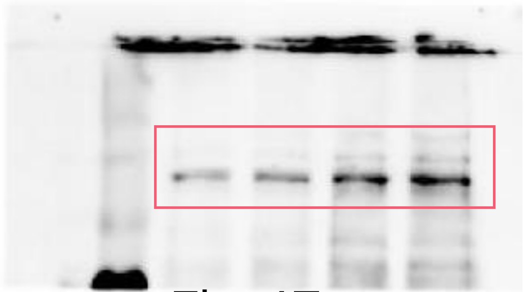

Fig. 4E
HepG2-p-PERK

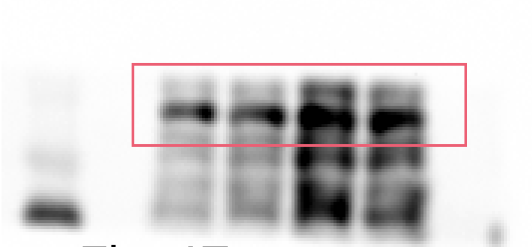

Fig. 4E
Huh7-p-PERK

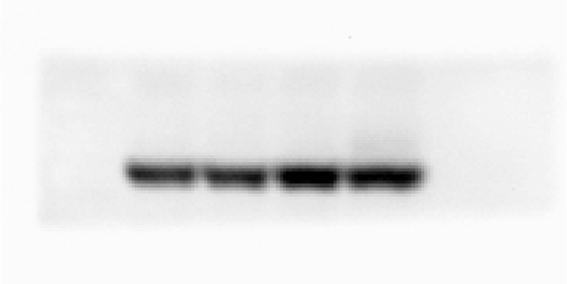

Fig. 4E
HepG2- ATF4

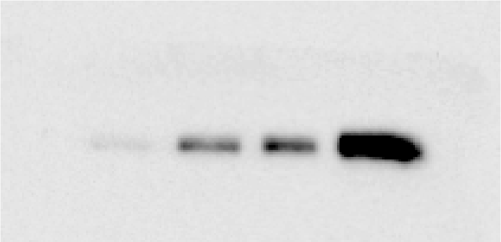

Fig. 4E
Huh7- ATF4

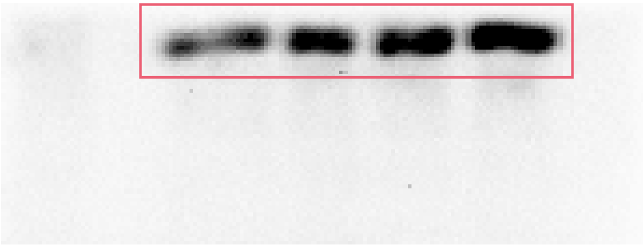

Fig. 4E
HepG2-p-eiF2α

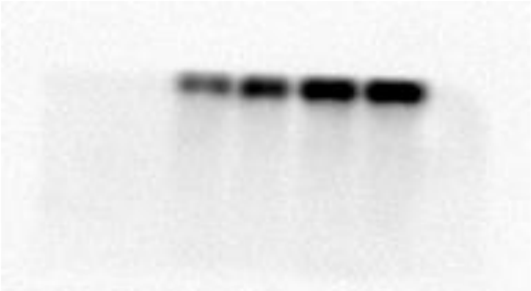

Fig. 4E
Huh7-p-eiF2α

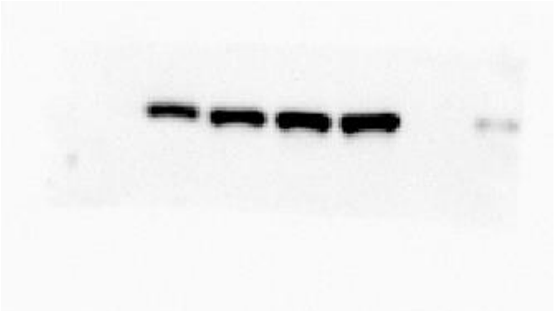

Fig. 4E
HepG2- CHOP

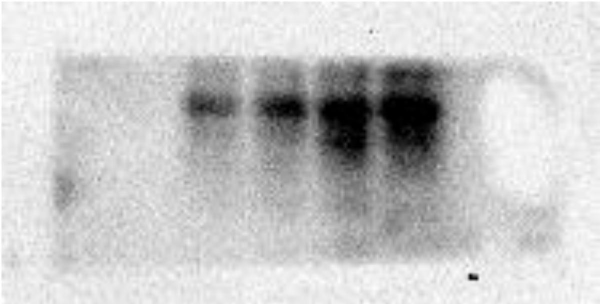

Fig. 4E
Huh7- CHOP

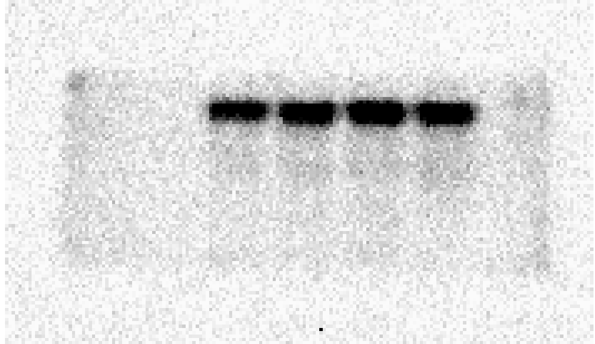

Fig. 4E
HepG2- eiF2α

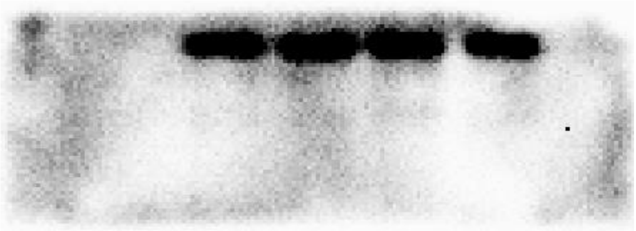

Fig. 4E
Huh7- eiF2α

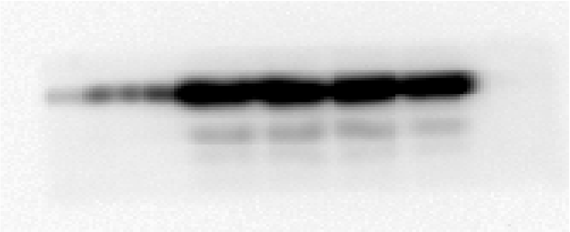

Fig. 4E
HepG2- GAPDH

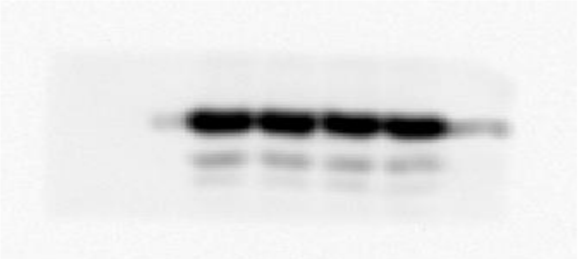

Fig. 4E
Huh7- GAPDH

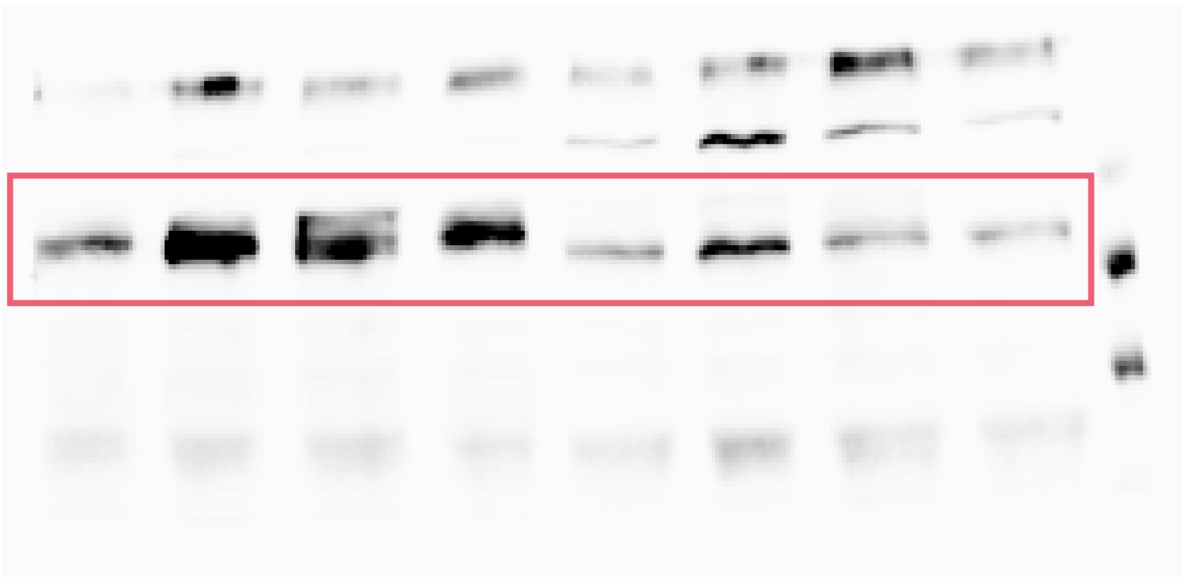

Fig. S5
HepG2-porimin

Fig. S5
Huh7-porimin

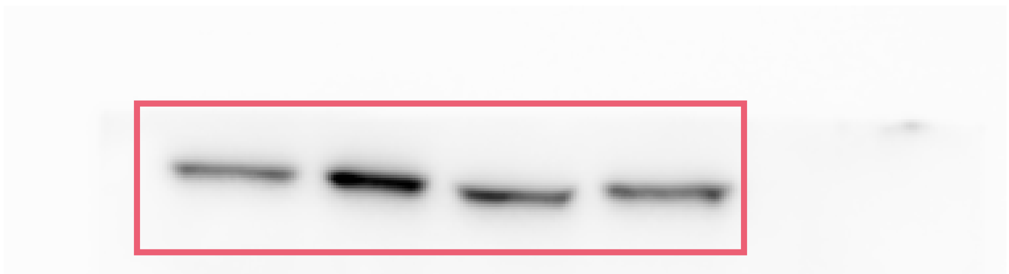

Fig. S5
HepG2-ERO1

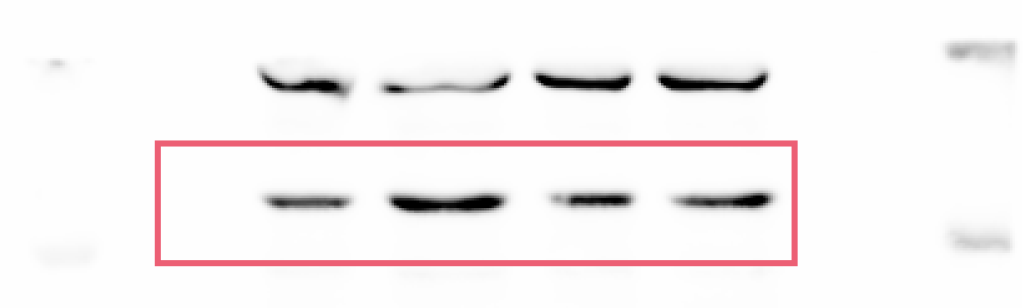

Fig. S5
Huh7-ERO1

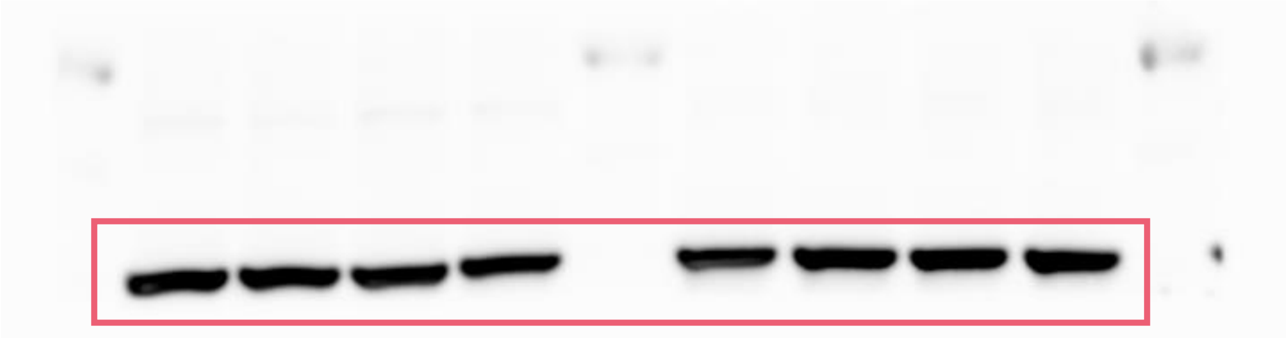

Fig. S5
HepG2-GAPDH

Fig. S5
Huh7-GAPDH

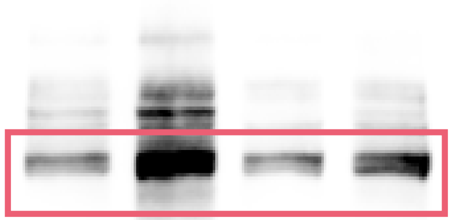

Fig. S6
HepG2-p-PERK

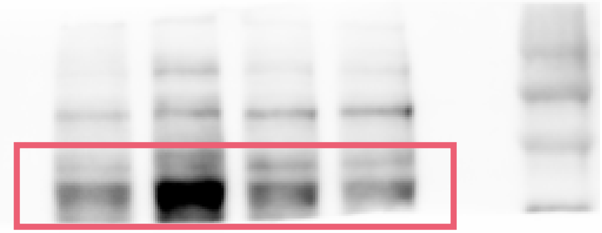

Fig. S6
Huh7-p-PERK

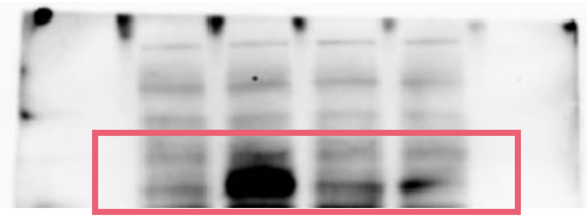

Fig. S6
HepG2- ATF4

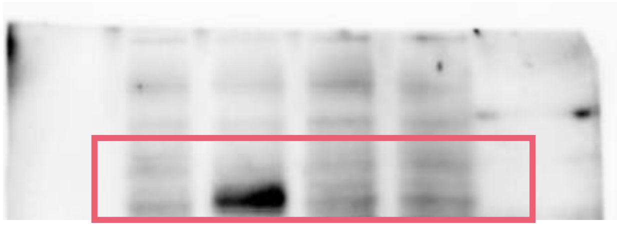

Fig. S6
Huh7- ATF4

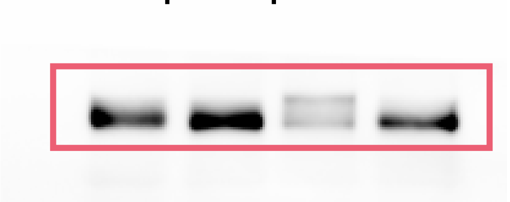

Fig. S6
HepG2-PERK

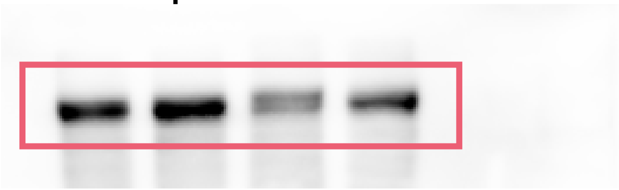

Fig. S6
Huh7-PERK

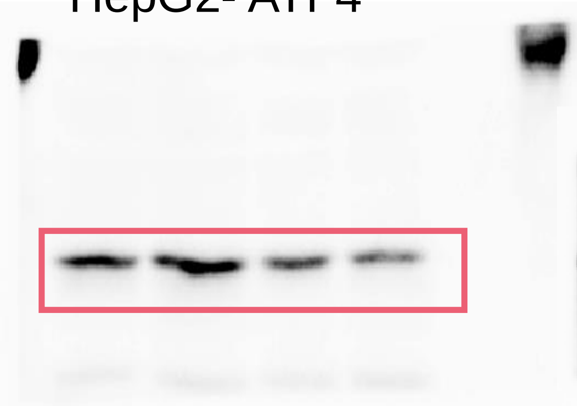

Fig. S6
HepG2- CHOP

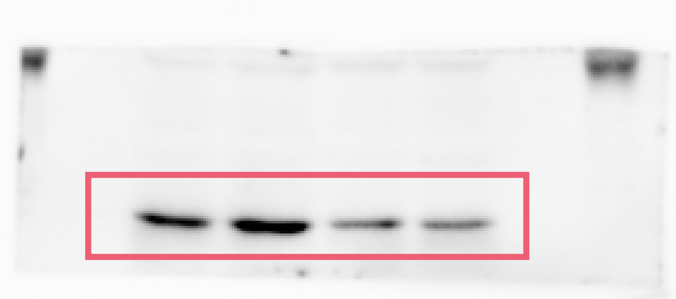

Fig. S6
Huh7- CHOP

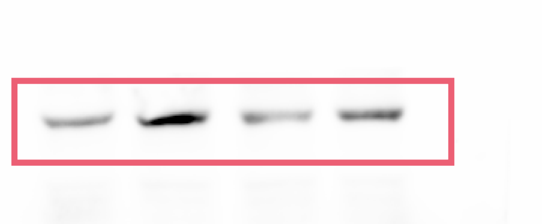

Fig. S6
HepG2-p-eIF2α

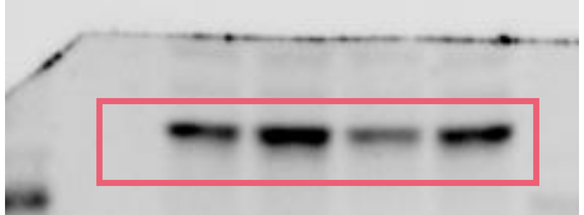

Fig. S6
Huh7-p-eIF2α

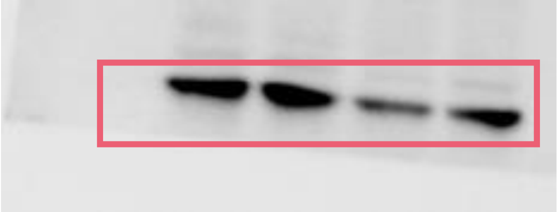

Fig. S6
HepG2-eIF2α

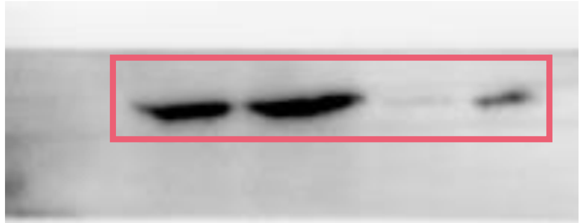

Fig. S6
Huh7-eIF2α

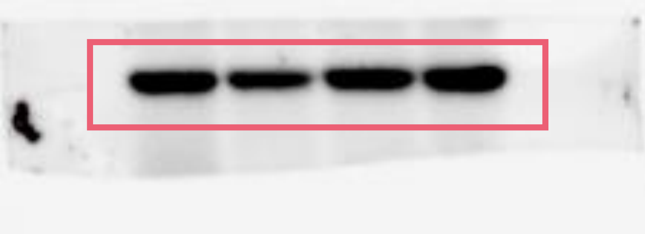

Fig. S6
HepG2- GAPDH

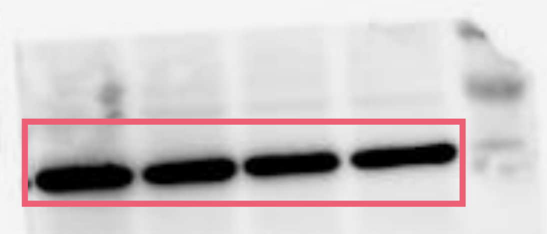

Fig. S6
Huh7- GAPDH

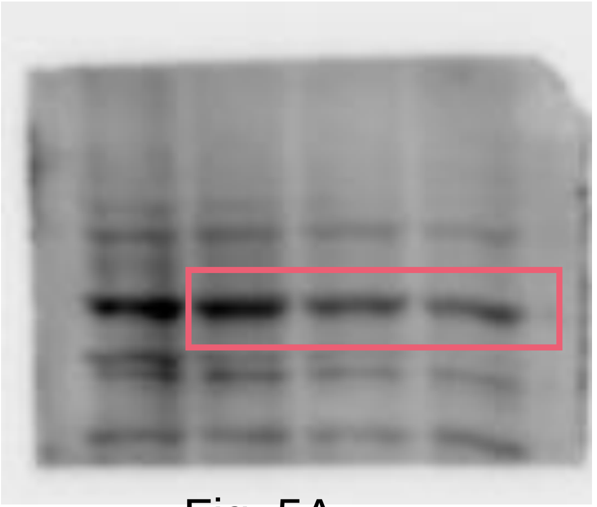

Fig. 5A
HepG2- ATF4

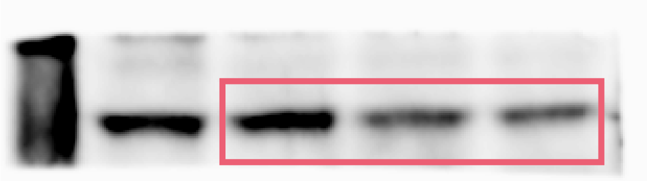

Fig. 5A
Huh7- ATF4

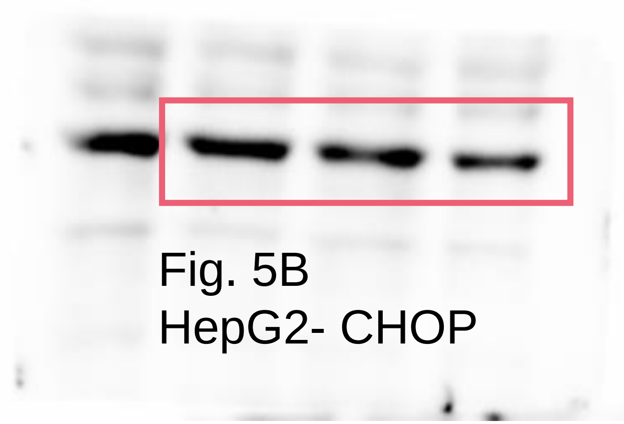

Fig. 5B
HepG2- CHOP

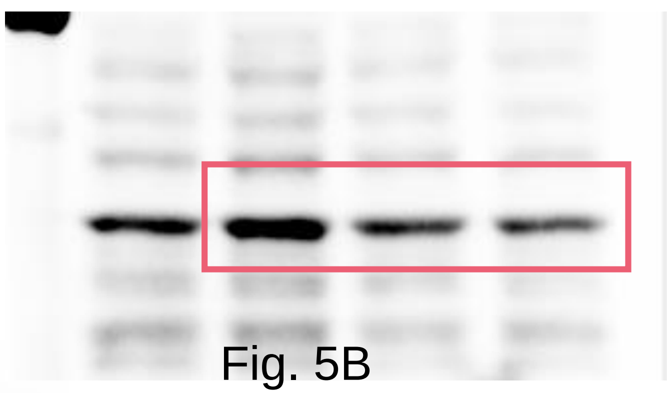

Fig. 5B
Huh7- CHOP

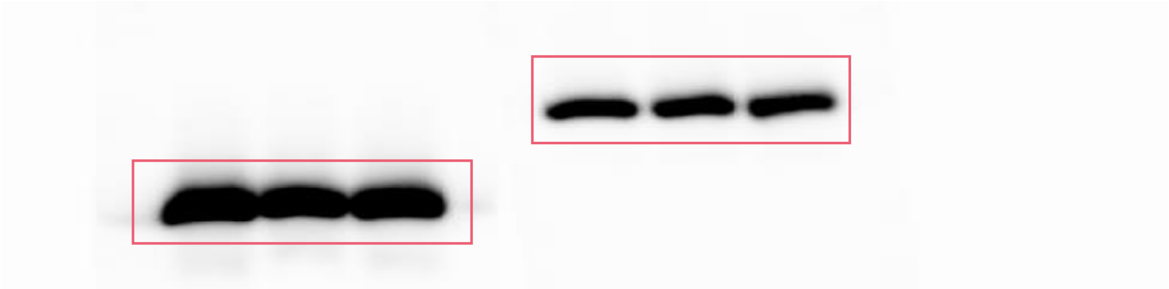

Fig. 5A
HepG2- GAPDH

Fig. 5A
Huh7- GAPDH

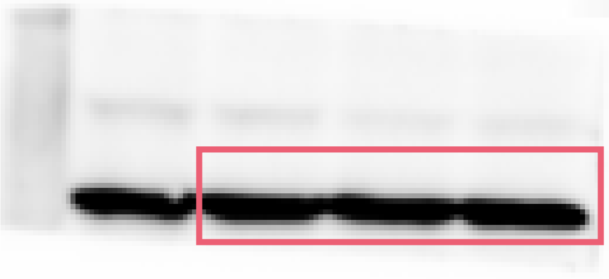

Fig. 5B
HepG2- GAPDH

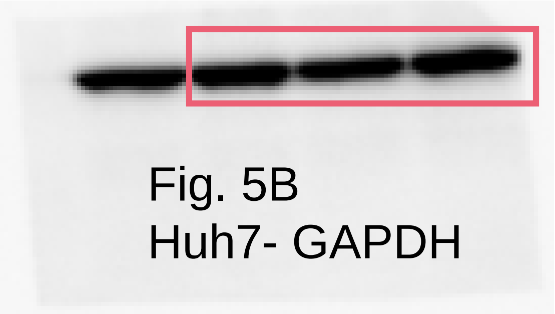

Fig. 5B
Huh7- GAPDH

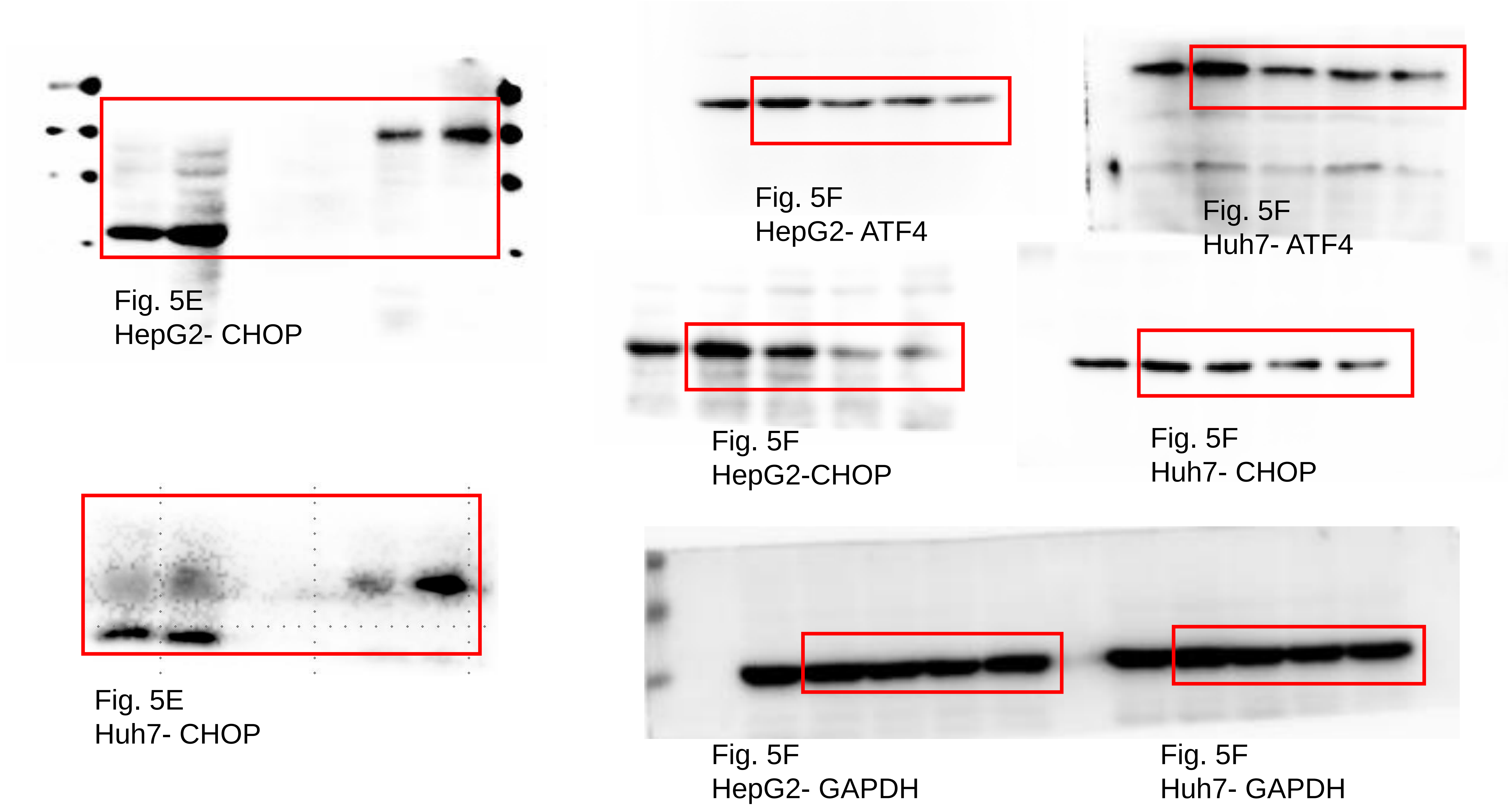
Fig. 5E
HepG2- CHOP
Fig. 5F
HepG2- ATF4
Fig. 5F
Huh7- ATF4
Fig. 5F
HepG2-CHOP
Fig. 5F
Huh7- CHOP
Fig. 5E
Huh7- CHOP
Fig. 5F
HepG2- GAPDH
Fig. 5F
Huh7- GAPDH

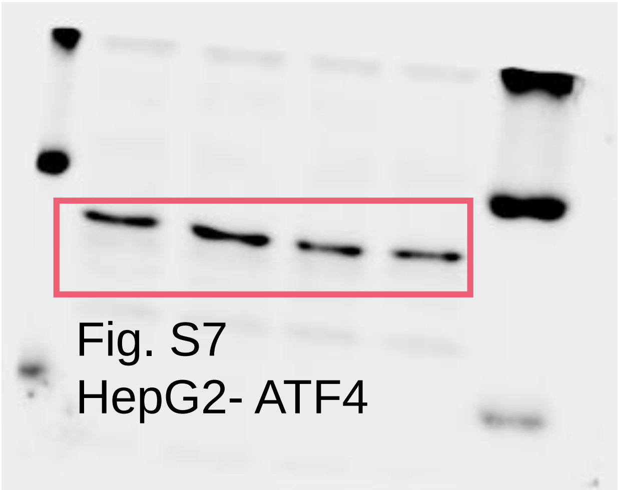
Fig. S7
HepG2- ATF4

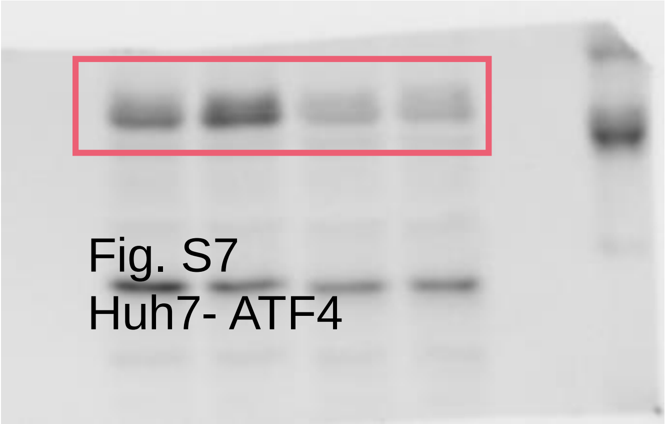
Fig. S7
Huh7- ATF4

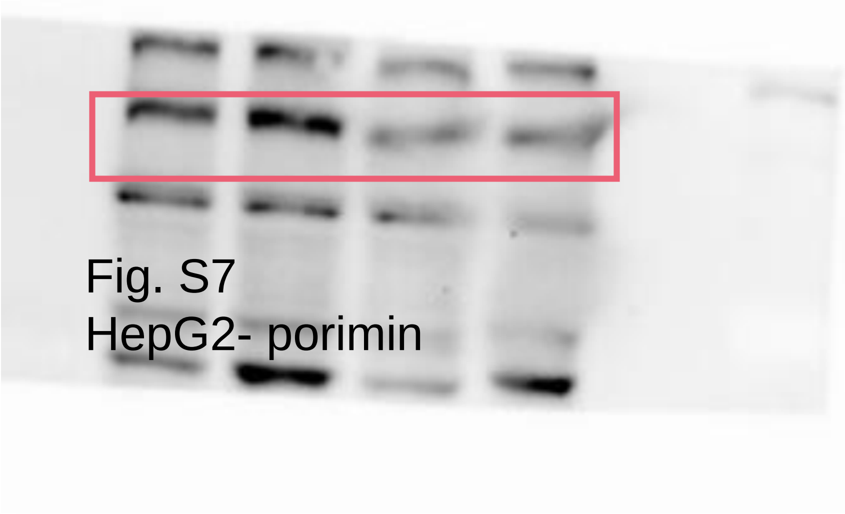
Fig. S7
HepG2- porimin

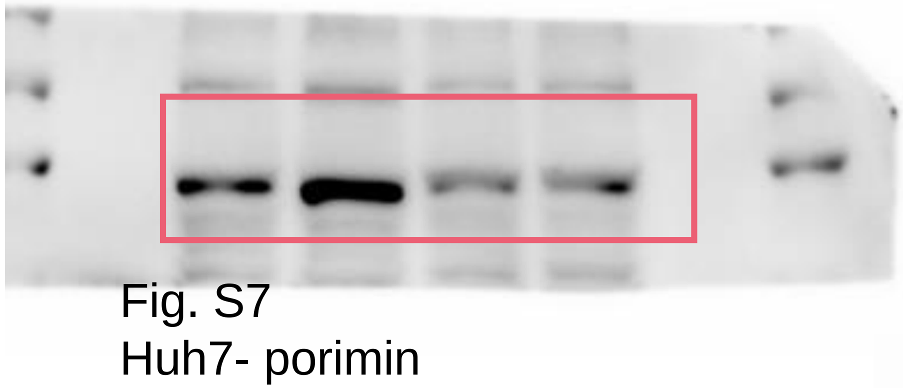
Fig. S7
Huh7- porimin

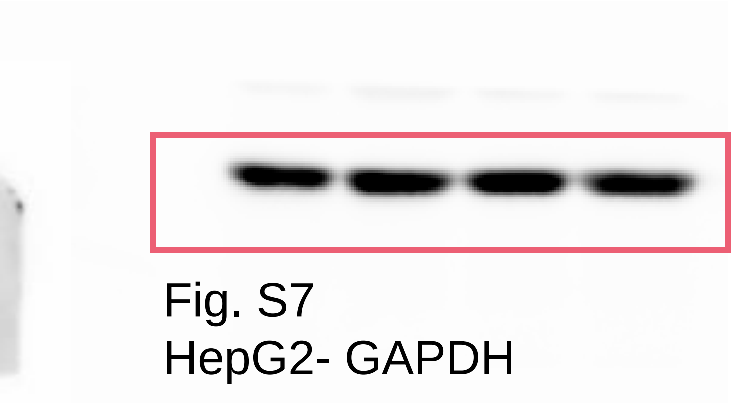
Fig. S7
HepG2- GAPDH

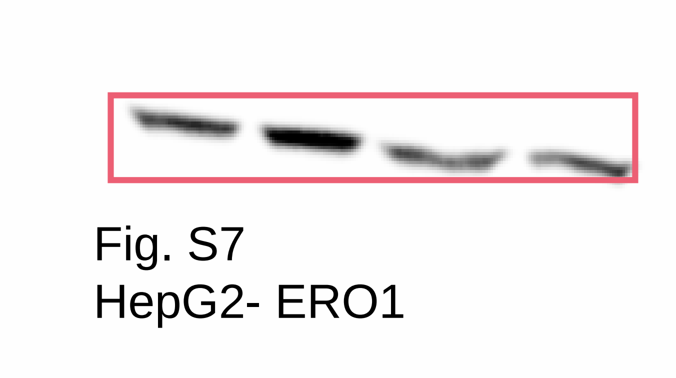
Fig. S7
HepG2- ERO1

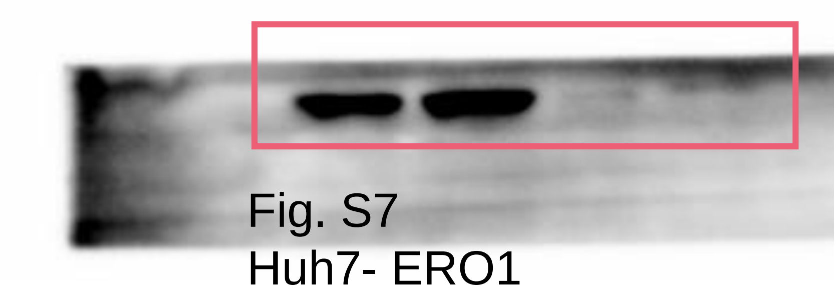
Fig. S7
Huh7- ERO1

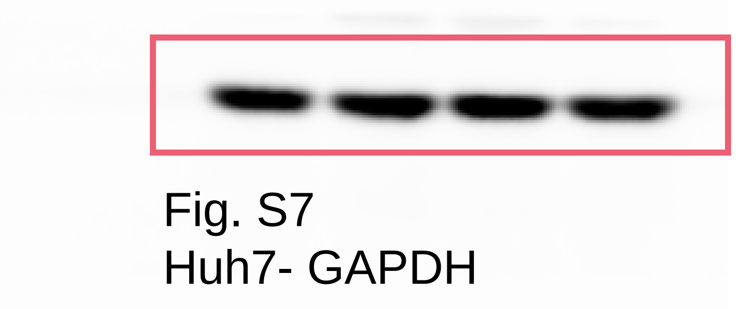
Fig. S7
Huh7- GAPDH

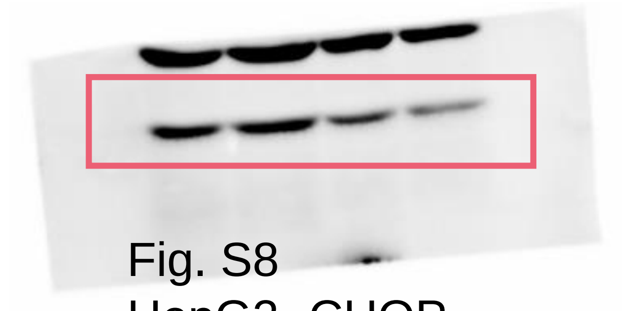

Fig. S8
HepG2- CHOP

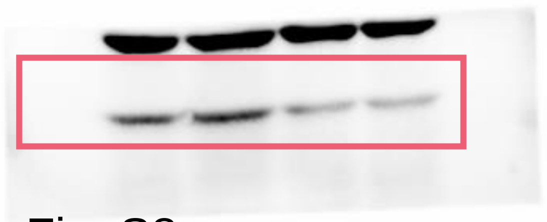

Fig. S8
Huh7- CHOP

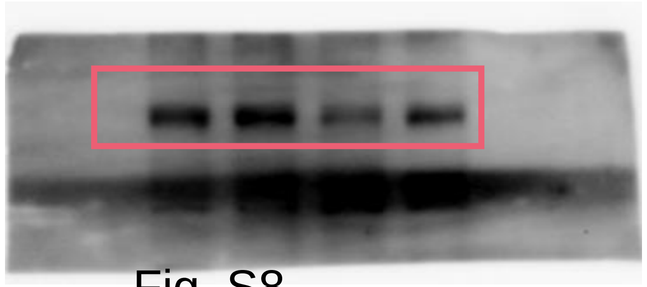

Fig. S8
HepG2- porimin

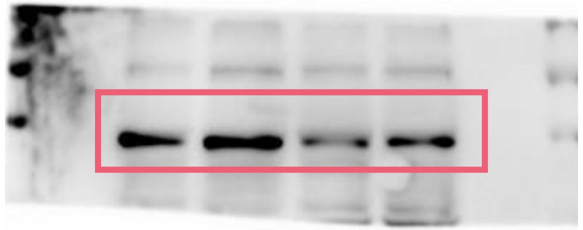

Fig. S8
Huh7- porimin

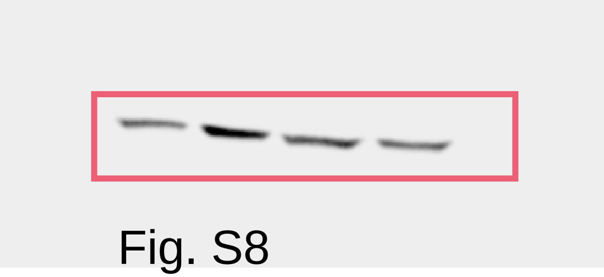

Fig. S8
HepG2- ERO1

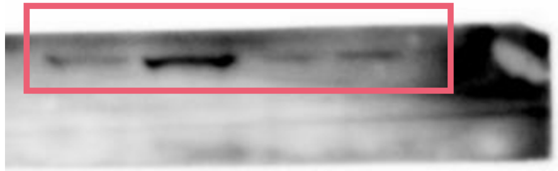

Fig. S8
Huh7- ERO1

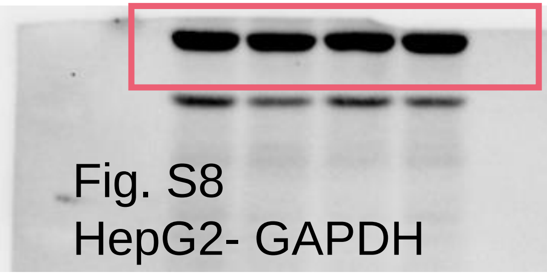

Fig. S8
HepG2- GAPDH

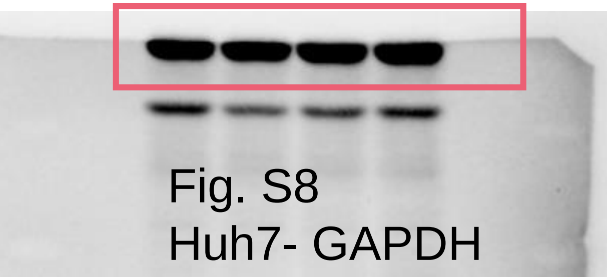

Fig. S8
Huh7- GAPDH
